# Jumping to attributions during social evaluation

**DOI:** 10.1038/s41598-024-65704-y

**Published:** 2024-07-04

**Authors:** Isabel H. W. Lau, Jessica Norman, Melanie Stothard, Christina O. Carlisi, Michael Moutoussis

**Affiliations:** 1 MRC Cognition and Brain Sciences Unit, University of Cambridge, Cambridge, UK; 2 Institute of Cognitive Neuroscience, University College London, London, UK; 3 Division of Psychology and Language Science, University College London, London, UK; 4 Department of Imaging Neuroscience, University College London, London, UK

**Keywords:** Cognitive neuroscience, Computational neuroscience, Social behaviour

## Abstract

Social learning is crucial for human relationships and well-being. Self- and other- evaluations are universal experiences, playing key roles in many psychiatric disorders, particularly anxiety and depression. We aimed to deepen our understanding of the computational mechanisms behind social learning, which have been implicated in internalizing conditions like anxiety and depression. We built on prior work based on the Social Evaluation Learning Task (SELT) and introduced a new computational model to better explain rapid initial inferences and progressive refinement during serial social evaluations. The Social Evaluation Learning Task-Revised (SELT-R) was improved by stakeholder input, making it more engaging and suitable for adolescents. A sample of 130 adults from the UK completed the SELT-R and questionnaires assessing symptoms of depression and anxiety. ‘*Classify-refine*’ computational models were compared with previously successful Bayesian models. The ‘*classify-refine*’ models performed better, providing insight into how people infer the attributes and motives of others. Parameters of the best fitting model from the SELT-R were correlated with Anxiety factor scores, with higher symptoms associated with greater decision noise and higher (less flexible) policy certainty. Our results replicate findings regarding the classify-refine process and set the stage for future investigations into the cognitive mechanisms of self and other evaluations in internalizing disorders.

## Introduction

Generalized anxiety, social anxiety, and depression are among the most prevalent mental health conditions encountered in both community and primary care settings. According to the World Mental Health Survey, approximately 25% of people worldwide have or have had an anxiety disorder, and 17% encounter depressive disorders at some point in their lives^1^. However, existing treatments are not effective for everyone who suffers^2,3^. There is therefore a pressing need to understand the cognitive mechanisms underpinning symptoms of anxiety and depression so that we can improve prevention and treatments.

Evaluating ourselves and other people is a universal experience, and differences in the way individuals evaluate themselves and others are central to many psychiatric disorders, including depression and anxiety. Given the shared social-cognitive mechanisms across internalizing disorders^4–6^, understanding social learning as a transdiagnostic mechanism across anxiety and depression is pivotal. In social anxiety, individuals worry a lot about others judging them negatively. A similar self-criticism is seen in depression. Generalized anxiety entails pervasive worry, social anxiety involves fear of negative evaluation in social situations, and depression is marked by persistent sadness and negative self-views, potentially linked to reduced sensitivity to positive social feedback^4,5,7–9^. While the underlying biopsychosocial and computational mechanisms have been investigated, improved validity and reliability of cognitive tasks, as well as better-fitting models to explain behavior on such tasks, are urgently needed to track the development of these disorders and to target interventions^4–6^.

**Figure 1:**
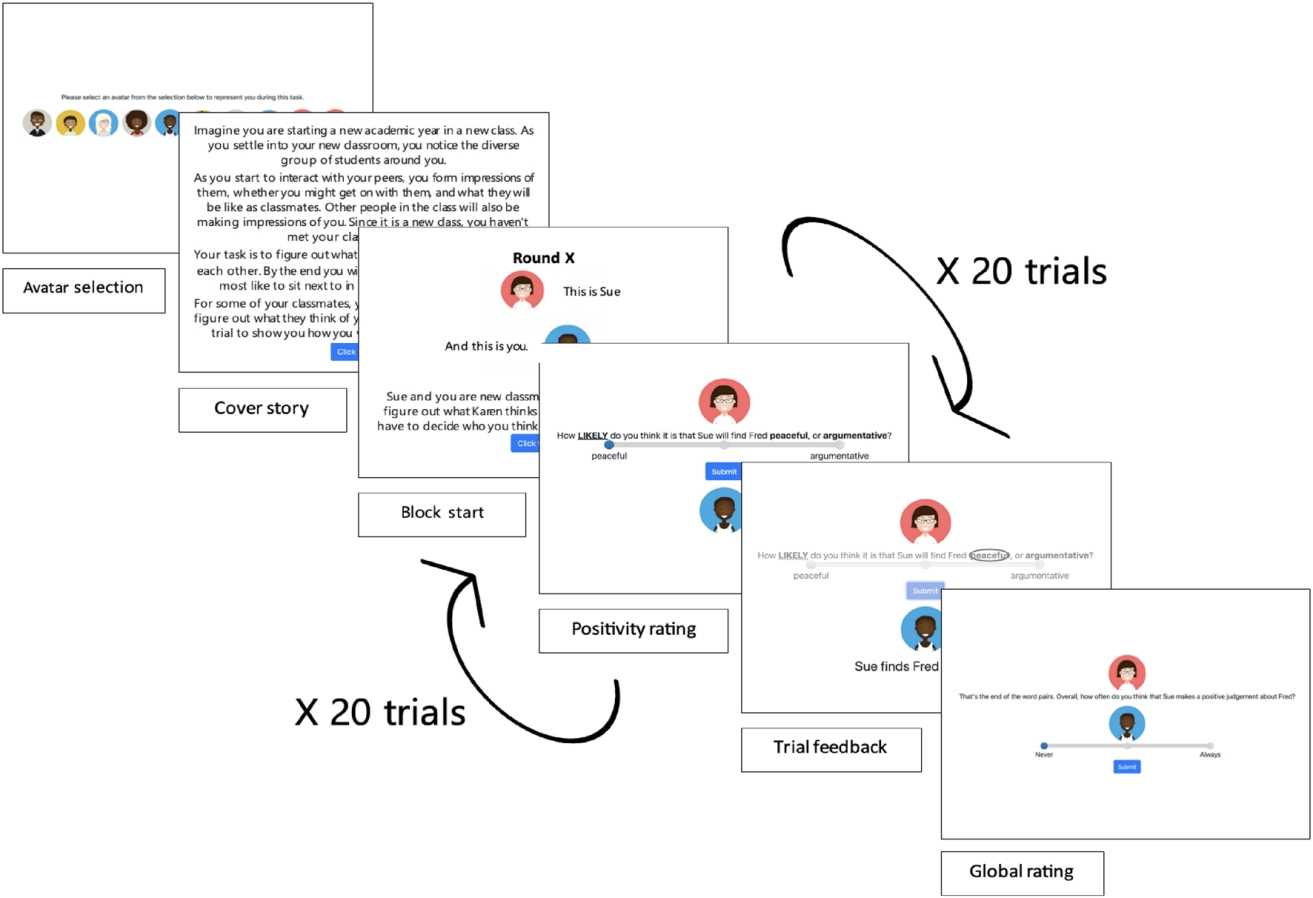
Example of a self-referential rule block in the SELT. Participants first chose an avatar to represent themselves and were given the cover story to illustrate the task context. Each block contains a learning phase with 20-word pairs and feedback, followed by a global rating phase. There was 1 practice trial (no feedback), and then 8 blocks: self-liked, self-liked-repeated, self-neutral, self-disliked, other-liked, other-liked-repeated, other-neutral, and other-disliked. For each trial, participants were required to provide a positivity rating, indicating their certainty of the rater’s choice, and were provided with feedback (e.g., whether the rater liked or disliked them in the self-referential condition, or another ratee in the other-referential condition) right after their ratings. Positive word accuracy varied: 0.8 for like and liked-repeated, 0.5 for neutral, and 0.2 for dislike.

The Social Evaluation Learning Task (SELT) has been successfully used to probe these mechanisms^10^. Here, participants observe ‘raters’ evaluating ‘ratees’. At each trial, they have to predict whether the current rater will evaluate the ratee positively or negatively. Ratees can be either the participant themselves, or a third party, and receive feedback from the ‘rater’ for subsequent learning (see Fig. 1). In the context of social learning, associative learning models were first used to understand how individuals establish connections between social outcomes, that is, a history of approvals and disapprovals, and their subsequent responses^11^. These models encompass parameters such as learning rates, which dictate the speed at which associations between stimuli and social rewards or social punishments develop, and positivity biases which reflect baseline expectations. Using such models, researchers have started to elucidate differences in cognitive mechanisms depending on psychopathology. One study found that adolescents with more severe depressive symptoms tended to display a reduced positive bias regarding social evaluation, suggesting a diminished inclination to view situations optimistically^11^. Social anxiety is a risk factor for subsequent generalized anxiety and depression, contributing to the maintenance of their symptoms. Consistent with this, depression may result from reduced sensitivity to social feedback^4,12,13^, on top of reduced positive biases.

While findings about reduced self-positive bias in psychiatric research clarify how individuals make decisions and learn in social contexts, Rescorla-Wagner associative models do not account well for the role of initial expectations and uncertainty during learning. Initial expectations, influenced by self-schemas that shape self-perception in social situations (e.g., thinking “I'm a bad person because no one befriends me”), are subtler than a simple decision-oriented bias used in associative models^14^. Additionally, lack of complete certainty in beliefs regarding social feedback (e.g., “I am 80% certain that John dislikes me”), is important for social learning and poorly approximated by associative models. Bayesian models based on evidence accumulation (‘*beta-belief*’ models) have quantified the positive and negative evidence in self- and other-schemas, explaining positive biases, uncertainty, and learning rates^14^. ‘*Beta-belief*’ models have given a useful account of social learning in social learning tasks, including the SELT^14–16^. Their parameters can differentiate between healthy participants, and those with high levels of mood and social anxiety symptoms^11,14,17^.

However, our previous work showed a need for improvements in both the SELT and its computational models^14,17,18^, as they have required large samples and sophisticated statistical analyses to demonstrate effects^17^. Task models did not fully address the rapid initial learning of evaluations, and later progressive refinement, highlighting the need for new models. In addition, the SELT is suboptimal for use with young people, because of the vocabulary used and its long, repetitive nature. This is crucial, as anxiety and depression most commonly emerge in adolescence^4,6^.

To overcome these limitations, we introduced a revised version of the SELT, the SELT-R, and a new modeling framework. This framework considers both fast initial learning and subsequent gradual refinement. The SELT-R consisted of eight 20-trial blocks, introducing “self-liked-repeated” and “other-liked-repeated” blocks. SELT-R’s eight distinct blocks, covered all 8 combinations of referential (i.e., self-, other-), and feedback (e.g., liked, liked-repeated, neutral, disliked) conditions: self-liked, other-liked, self-liked-repeated, other-liked-repeated, etc. In the self-referential blocks, participants were asked to make a judgment about how likely the rater was to provide positive feedback about themselves (e.g., participant's chosen avatar/'ratee'), whereas in the other-referential blocks, participants had to judge how likely the rater was to provide positive feedback about another peer (i.e., another computerised ‘ratee’ that is not the participant's). In the liked and liked-repeated feedback blocks, the likelihood of positive words being accurate was set at 80% (i.e., 16 trials each), whereas in 'disliked' blocks, it was 20% (i.e., 4 trials). For 'neutral' blocks, the likelihood was 50% (i.e., 10 trials). Their order was carefully pseudo-randomized to follow the “disliked” rule conditions (see “Methods” for more details of the task), for a better assessment of the impact of negative expectations in subsequent social learning. Within each trial, participants were presented with pairs of personality trait descriptors (one negative and one positive) and asked to rate the probability (ranging from 0 to 100) of the rater selecting the positive word in each trial. Higher ratings indicated a participant’s belief that the rater is likely to provide positive feedback, while lower ratings indicated an expectation of negative feedback. After the participant made their rating, the correct word was circled, according to the block rule (i.e. liked, neutral, or disliked).

To improve the ecological validity of the SELT, we gathered stakeholder feedback through focus groups. Based on the focus group findings, we implemented changes, to make the task more engaging to adolescents. Specifically, we asked young people for their suggestions as to whether it was easy to understand and engaging, and whether the pairs of positive–negative attributes in the task were clear (see “Results”—Focus group findings).

**Figure 2:**
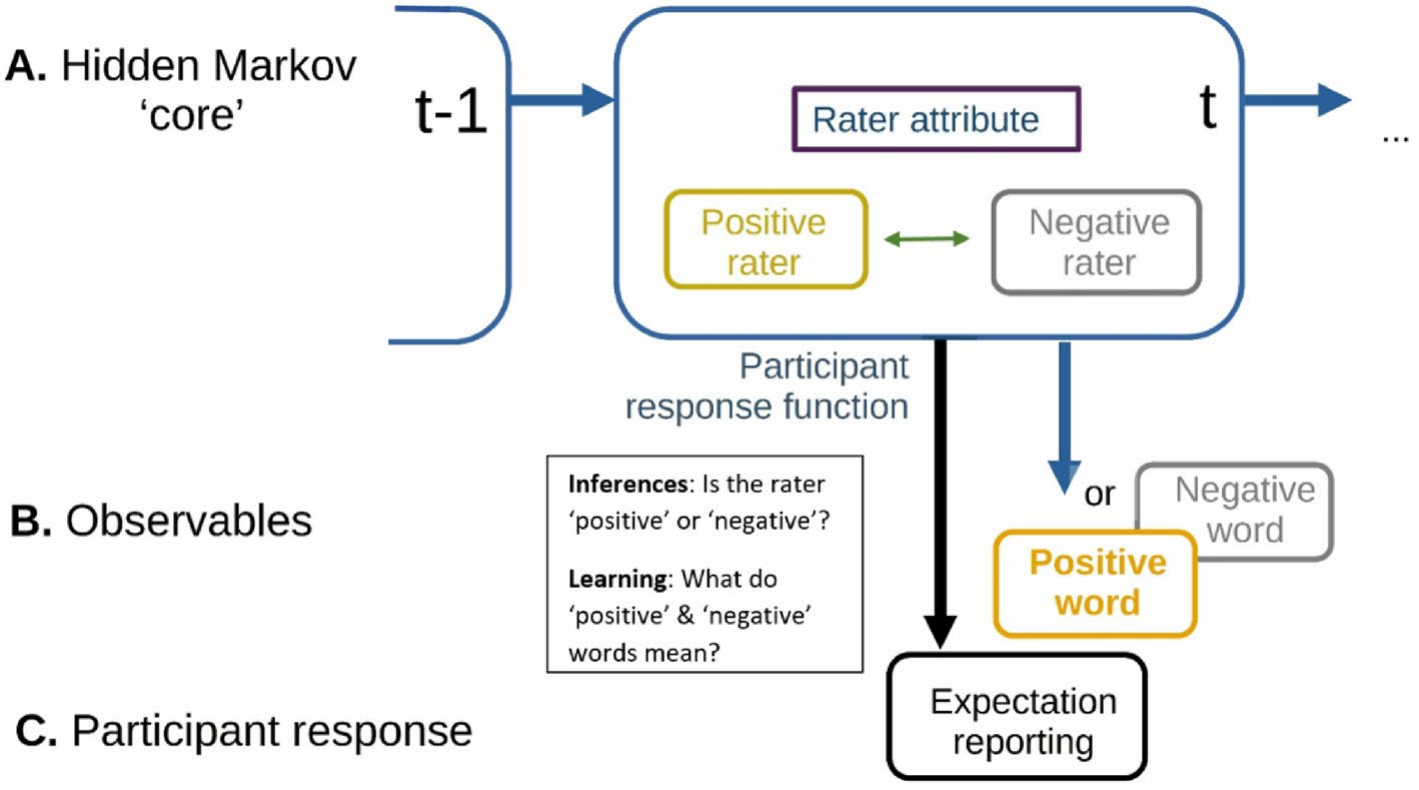
Task-related inferences and responses model. The diagram shows how participant response is generated based on their unobserved internal model across two trials. **Row A**: Participants hold a model of raters. This comprises beliefs about how positive or negative raters are about the ratee. Here, the Hidden Markov ‘core’ (an unobservable internal model) is especially valuable in modeling sequential data. The concealed state of a system (e.g., determining whether the rater is positive or negative) remains unobservable but can be inferred through the examination of observed data points over the trials, *t*. **Row B**: Participants observe raters give positive or negative, but always categorical, ratings at each trial. They assume that these ratings may not directly reflect underlying beliefs, but may be, for example, subject to desirability biases. They update their beliefs based on these observations. **Row C** Participants reported how likely they thought the rater was to provide a positive response. Again, this is not a direct representation of Rater beliefs, but could be subject to biases of either the Rater or the participant (‘participant response function’).

In our pursuit of a deeper understanding of the cognitive mechanisms of social evaluation, we considered the concept of *black-and-white thinking*, where individuals may form polarized beliefs about themselves and others^19^. Such concepts are often used to explain unhelpful attributions in cognitive-behavioral as well as psychodynamic models of internalizing disorders, such as ‘splitting’ (e.g., polarizing views into “all good” or “all bad” categories). In Cognitive Behavioral Therapy, *black-and-white thinking* is a cognitive bias, whereby a person tends to perceive things or people as all good or all bad, without acknowledging the gray areas between^19–21^. Hence, we hypothesized about SELT's capability to correlate with internalizing symptoms and its demonstrating rapid initial inferences, in terms of *black-and-white thinking*^19^. This led to the introduction of novel *'classify-refine*' models of SELT, which could account for rapid initial learning (i.e., inference), whose rate subsequently declined^18^ (see Fig. 2). Recent evidence supports this approach in a different attributional task^15,18^. Here, we contrasted '*classify-refine*' with established ‘*beta-belief*’ models, and compared model fit in the SELT-R. Our hypotheses were that:

1. People first make rapid judgments of whether others evaluate a person positively or negatively—a process of initial classification rather than gradual learning.
2. Individuals with higher internalizing symptom scores would show different social learning mechanisms, which would be captured by the novel ‘*classify-refine*’ model.

To test these hypotheses, we developed an optimized ‘*classify-refine*’ model and compared it against a comparably optimized ‘*beta-belief*’ model^14,18^.

The SELT-R employed in this study is tailored for young individuals. To revise and improve the task, we conducted two focus group sessions to collect feedback from groups of young individuals regarding the design of the task, and subsequently revise the task to make it more youth-oriented (see “Methods”). However, we initially tested hypotheses regarding task performance and model parameters in an online adult sample, before conducting upcoming large-scale studies that will involve testing in young people. We acknowledged that adults and young people might have different perceptions of feedback (e.g., different salience of feedback words). Indeed, some studies show learning differences between adolescents and adults^22^. Nevertheless, task instructions and words used were carefully phrased so that they are linguistically more accessible to populations with a simpler vocabulary (e.g., using ‘caring’ instead of ‘compassionate’ so that it is more understandable by young individuals) (see Table S2). While our primary interest lies in understanding adolescents, we took an iterative approach by first conducting model comparisons in adults, as much of the initial work on this task and models has been done with adult populations. This allows us to establish a foundation before translating the work to adolescent samples as a next step. We recruited 130 participants from the UK online on Prolific^23^ measured symptoms of depression, anxiety, and social anxiety using self-report measures including the Generalized Anxiety Disorder 7 (GAD-7)^24^, Patient Health Questionnaire 8 (PHQ-8)^25^, State-Trait Anxiety Inventory—Trait version (STAI-Trait)^26^, and the Social Anxiety and Depression Scale for Adolescents (SMSAD-A)^27^. Given the overlapping socio-cognitive mechanisms in internalizing disorders, we used questionnaires that are short, easily accessible, and of good psychometric properties in measuring social anxiety, general anxiety, and depression. The GAD-7, PHQ-8, and STAI-Trait were chosen as they are widely used questionnaires with good psychometric properties in capturing general anxiety (symptoms and traits) and depression^25,26,28^. SMSAD-A was chosen as it provides a short and accessible measure of social anxiety^29,30^.

In addition, a factor analysis was performed to investigate the commonality across symptom self-report measure scores. Here, factor analysis helped us derive more reliable, less noisy measures by consolidating similar questionnaires into a weighted average. Reducing the number of psychometric constructs that we relate to the computational model also helps reduce multiple comparisons. We assessed participants’ social learning using the modified SELT-R task. Computational analyses included model fitting and model comparison, which employed Bayesian Information Criterion (BIC) to assess model fit and select the best-fitting model (see “Methods”).

## Results

### Focus group findings

Given the prevalence of anxiety and depression in adolescence^4,12,31^ and the importance of social interactions during this developmental window, it is important to investigate the computational underpinnings of social learning in young people. We, therefore, wanted to adapt the existing SELT so that it can be used in adolescents in future research. To inform task modifications so that the SELT was more engaging for young people, we organized two focus groups with young people between ages 11 and 18 (*n* = 22), to evaluate its feasibility and acceptability.

After the feedback received from these focus groups, we made modifications to the previous SELT version^10^ (see Table 1). In the revised version (i.e., SELT-R), participants can customize their avatars, strengthening their connection with them. Additionally, we incorporated a cover story that sets the task in a school environment at the start of a new academic year. In this scenario, participants are asked to choose "classmates" (i.e., raters) that their avatar (i.e., in the self-referential condition) and another "classmate" being rated (i.e., the ratee's avatar in the other-referential condition) would prefer to sit next to at the end of the task, highlighting the task's social context. We also adapted word pairs based on young people’s feedback, ensuring that the vocabulary used was relevant and accessible to adolescent participants. A full summary of focus group findings can be found in the supplementary information (see Table S1).

**Table 1:** Focus group findings that directly informed task revision.

Focus group feedback	Proposed change and rationale
The task is too long and tedious The task lacked an overarching aim	Task block length reduced Added back story of meeting new classmates to make the task relatable and engaging
Several words were flagged by participants as being difficult to comprehend	Edited word pairs to be more developmentally appropriate Removed words that were ambiguously positive/negative (e.g. “*emotional*”)
Certain words were too similar or related in meaning (e.g. “*brave*”, and “*confident*”) within the same task block	Separated closely related words into different task blocks
Participants liked the inclusion of an avatar and felt that it made them immersed and invested in the task	Included the selection of an avatar to represent the participant at the beginning of the task
Participants were interested in how the ethnicity and/or gender of the rater might influence responses One participant thought that they might have received negative feedback because they had chosen an avatar with black skin	The avatars for both raters and ratees had a variety of characteristics, including skin color and gender

We employed changes, including adding a cover story, allowing participants to choose their avatars, and adapting more accessible word pairs for adolescents to make SELT-R more suitable for young people. Due to the constraints of the consent form and ethnic approval, we are not able to quote directly from the participants, and thus all qualitative findings are summarized according to themes.

### Actions taken and justification

We have acknowledged that some participants were interested in the effect of ethnicity on social learning. We were concerned about the role that identity issues may have played in our data. We therefore conducted an exploratory mixed-effect model to study the main effect of ethnicity to social learning on positive words for the self- and other-referential conditions. A main effect of ethnicity in the other-referential condition was found (see Table 2). Specifically, Muslim and Black ratees had higher positive ratings than White ratees. However, there were no main effect of ethnicity for self-referential condition ratee (*p* > 0.05), nor there are coalitional ethnicity effect (i.e., participants have different ratings when the ratees and rater are of the same ethnicity compared to different ethnicities) (*p* < 0.05). This suggests that there is a positive discrimination of non-White ratees to others.

**Table 2:** Mixed-effect model results for ethnicity of other-referential condition ratee.

	Estimate	SD	Error	df	*p*-value
Black	5.18	2.49	126.35	2.08	0.04
Muslim	6.39	2.75	31.85	3.32	0.03
White	1.83	2.03	118.76	0.90	0.37

### Demographics

The sample featured a well-balanced distribution of genders and was predominantly White British (mean age = 37.5*, *SD = 10.5). 44 (33.8%) Participants self-reported a current or historic diagnosis of depression or anxiety disorder, and 86.3% reported no additional neuropsychiatric diagnosis (86.3%). 91.5% had not used psychiatric medications in the past six months. Most participants fell within the middle-to-high socioeconomic status range (see Table S3).

### Descriptive findings

The distribution of positive ratings, both at the trial level and in the overall global context, was categorized across four distinct feedback conditions (disliked-, neutral-, liked-, and liked-repeated) and two referential conditions (self- and other-referential). Behavioral task responses showed notable differences between self- and other-referential conditions, positive ratings, and overall global ratings, indicating that individuals were adjusting their responses based on the feedback they received (see Table S4 and Fig. S1).

### Questionnaire results

Overall, participants exhibited modest psychological symptom scores (GAD-7: *M* = 6.3, SD = 5.5; PHQ-8: M = 7.0, *SD* = 5.8; and SMSAD-A: *M* = 8.3, SD = 9.3). The STAI-Trait showed moderate scores (*M* = 4.3, SD = 5.2) (Table S5 and Fig. S2). Thus, most participants had low internalizing symptom scores and moderate levels of trait anxiety, which aligns with expectations for a psychological trait in the general population^26^. Females, on average, displayed slightly higher scores in all questionnaire domains when compared to males (*p* < 0.001).

### Factor analysis results

To reduce dimensionality and increase sensitivity, we performed a factor analysis on the depression and anxiety trait and symptom scores. This resulted in two factors based on parallel analysis and scree plot (see Fig. S3). The first factor ‘Anxiety’ showed good stability and validity (factor loadings of STAI-Trait = 0.73, SMSAD-A = 0.79, and GAD-7 = 0.91). However, the second factor ‘Depression’, showed poor stability and validity (factor loading of PHQ-8 = 0.33), and thus was excluded in subsequent analysis (see Tables S6 and S7).

### Computational modeling

We compared models according to a hypothesis-based hierarchy. We examined two classes of models: The new ‘*classify-refine*’ models (see descriptions Table 3) vs. our previously successful ‘*beta-belief*’ models. First, we used model comparison to select the best model in each class. Then, we compared the winners across the classes. We then performed tests on simulated data to examine the adequacy of the best model to capture specific data features. Next, we examined the posterior correlations between task parameters and sought to reduce the dimensionality of parameter sets. Finally, we examined, in an exploratory manner, whether task measures, both computational parameters and descriptive statistics, accounted for internalizing symptoms.

**Table 3:** Step-by step description of the ‘Classify-refine’ model.

Stage	Information processing	Example	Key parameter(s)
1	Belief about what a ‘positive’ or ‘negative’ rater is	Participants believe that positive raters give positive words 60% of the time, and negative raters 10% of the time	Initial values of the likelihood map, A, that participants believe raters have (Supplement 1.1.i) Initial belief of positive attribution of the self (*pS*+) and the other (*pO*+)
2	Prior expectations about the state of raters (upon encountering one)	Participants formulate beliefs (in terms of effective evidence) of whether the rater will be positively or negatively disposed of	
3	Translation of the prior expectations into mean positivity (of positive and negative states) of the rater	Participants take account of their beliefs above, so they may expect on average 35% positive responses (e.g., if positive words are provided 60% in positive and 10% in negative states, and positive and negative states each occur half of the time)	
4	Reporting mean positivity, corrupted by decision noise and lapses	In our example, the 35% expected positivity is mapped to a Visual Analogue scale, and corrupted by a temperature-like decision noise and a uniform lapse rate	Decision precision (*αPrec*) Noise floor parameter (*lps*)
5	Observation of the actual outcome	Participants observe a response from the rater, for example, a positive word at a given trial	
6	Fast update of belief according to approximate Bayesian inference	Participants update their beliefs according to the response of the rater. Here, 6 out of 11 occurrences overall would be positive, and 5 out of 11 occurrences, negative. Note that the updates are not simply counts of outcomes, but include the ‘effective counts’ of prior expectations This stage is the 'classify' stage. The attribution (prior) certainty impacts the change in belief probability regarding the rater. For example, changing from 5 out of 10, to 6/11 impacts the probability more than from 50/100, to 51/101	Attribution Certainty (*dEv*)
7	Gradual learning about the present rater- Update of beliefs of Stage 1 above	Participants ‘refine’ their beliefs about raters In our example, observing a positive response increased the belief that a rater will give a positive response, and this is applied more strongly to a positive rater (who is more likely to be present, given the participant’s updated beliefs) than a negative one	Policy certainty (*aEv*)
8	Updating beliefs about the kind of raters one can encounter in the task	The participant thinks ‘OK, this block most likely had a positive rater. Hence, I must update my beliefs about the prevalence of positive raters in this task, and hence my expectations about the next block’	Learning parameters for regular (*λgen*) and repeated (*λrep*) blocks

#### Refined learning accompanies fast classification

**Figure 3:**
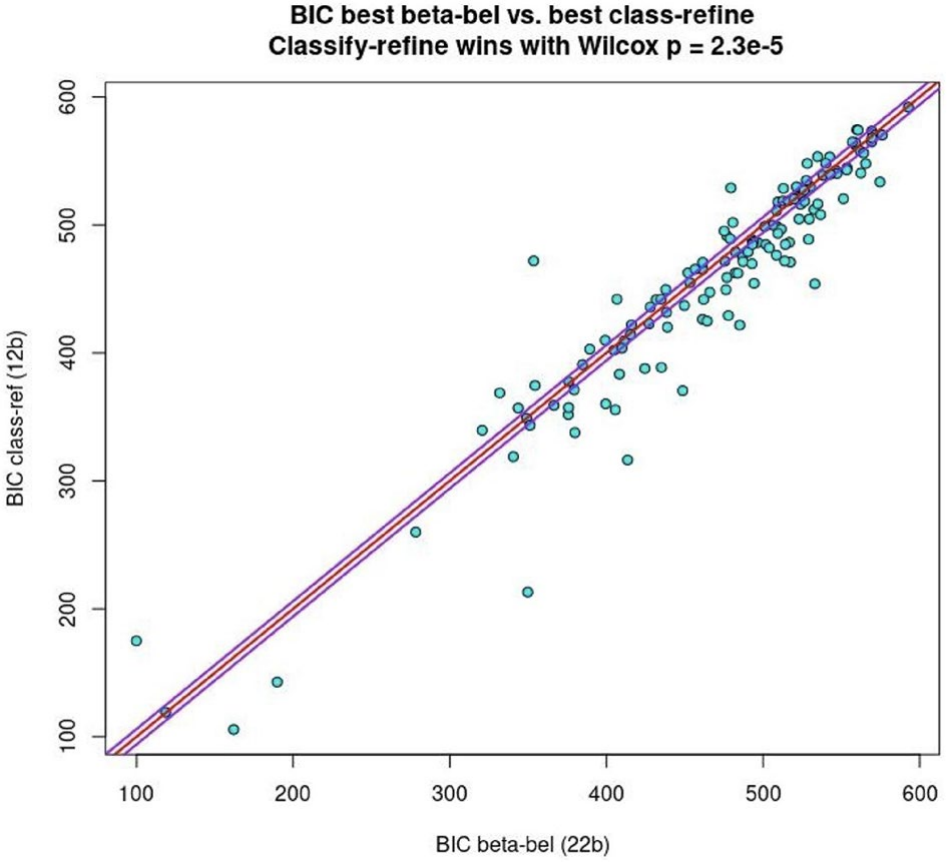
Key model comparison results. Red line = BIC equality. The winning ‘*classify-refine*’ model outperforms the best ‘*beta-belief*’ one. For 53/130 participants, the BIC difference is > 6 (i.e. to the right of the purple line of difference + 6). 28/130 participants had a BIC difference < −6 (left of purple lines). Hence the ‘*classify-refine*’ model describes behavior in the overall population better, Wilcoxon *p* = 2.3e−5.

We compared the winning ‘*classify-refine*’ model with the best ‘*beta-belief*’ model, concerning total BIC. The winning ‘*classify-refine*’ model outperformed the winning ‘*beta-belief*’ model (see Fig. 3) despite its slightly greater complexity. The total BIC value for the ‘*classify-refine*’ was 62,472 and for the ‘*beta-belief*’ 63,746. A paired Wilcoxon test was used to compare the two models and showed significant evidence in favor of the ‘*classify-refine*’ model, *p* = 2.27e−5.

**Figure 4:**
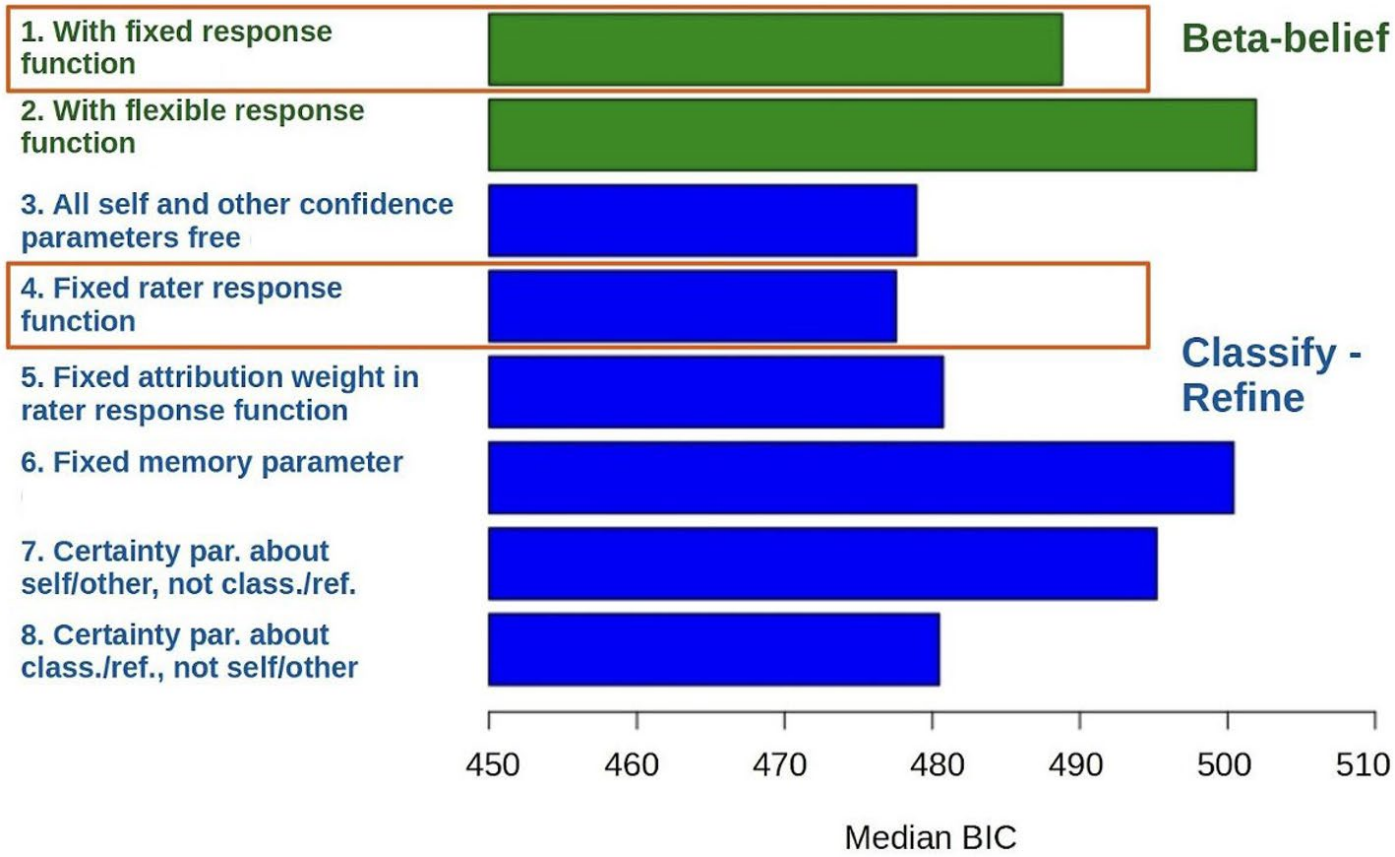
Key model comparison in terms of median BIC per participant. The models compared in detail in Fig. 3***.*** are framed in orange.

All models had, as a minimum, self- and other-positivity, attribution certainty, and decision noise (model parameters 1, 2, 3 and 5 in Table 4). More parameters were then introduced, fitted either freely to each individual, or subsequently fixed (weight of attribution [*wAttr*], bias [*w0*]) to the median value of the aforementioned free fit and applied to the whole population (see Table 4 [right-most column], Fig. 4, and Supplement 1.5). To simplify a model, one can remove a free parameter from the equation or fix it to a predetermined value. For instance, in a linear regression, setting a coefficient to zero (i.e., removing the parameter) or fixing the term to a constant (i.e., to a predetermined value) implies no effect of a relevant predictor variable. When fixing a parameter, we approximated the optimal value with the median of individual estimates when the parameter was fitted freely, ensuring that the fixed value is informed by the data. We preferred the median estimate over other central tendency estimates (e.g., mean estimate) when fixing a parameter, because it is less sensitive to outliers and skewed distributions. This approach balances model simplicity with explanatory power, yielding more interpretable results.

**Table 4:** Meaning and role played by model parameters.

	Model parameter	Technical definition and abbreviation	Key roles
Positivity			
1	Initial belief of positive attribution of the self	Central tendency of initial belief of positive attribution about the self, *pS*+	Greater *pS*+ leads to greater initial expectation of positive attribution of the Self, but how persistent this is depends both on evidence encountered (rater decisions seen) and, crucially, the certainty (strength) with this belief is held (*dEv* below)
2	Initial belief of positive attribution of the other	Central tendency of initial belief of positive attribution about the other, *pO*+	Similar to *pS*+ described above, but for positive attribution of the Other
Certainty			
3	Attribution Certainty	Certainty of prior belief of positive attribution, formulated as the amount of initial evidence, *dEv*. Relevant in ‘classify’ learning	Greater *dEv* denotes increased confidence about positive attribution of the rater in both the Self and Other referential conditions. Smaller certainty *dEv* directly contributes to greater responsivity in response to feedback in both referential conditions
4.	Policy certainty	Initial certainty parameter, *aEv*, for the policy followed by a ‘positive’ or ‘negative’ rater, crucial for ‘refine’ learning	Greater *aEv* denotes increased initial certainty about the evaluation of the raters in both the Self and Other referential conditions. *aEv* determines how certain participants are about the raters' policies. The more certain they are, the less one additional piece of evidence (observation) shifts their belief as participants formulate the pattern (‘policy’) of the rater’s response. We found that only one such parameter is needed for Self and Other (e.g., high posterior correlation)
Decision noise			
5	Decision precision	Decision precision, or inverse-temperature, *αPrec*	The accuracy, *αPrec*, of each decision across different trials of the same rater. Fixed throughout the task
6	Noise floor parameter	Noise floor parameter (*lps*) accounting for lapses of attention, motor slips, etc. It accounts for the fact that people do not always make decisions according to task values	A higher *lps* implies a more error-prone or less reliable in decision-making or performance. A lower *lps* suggests a higher degree of reliability and consistency in their decision-making or performance
Learning & forgetting			
7	Learning parameter from one dictator to the next	Learning rate- like measure from one rater to the next, *λgen*	A higher *λgen* leads to the prior beliefs regarding each rater being influenced by the posterior estimates of the raters seen before. It can be thought of as a strength of belief that raters seen during the experiment will resemble each other
8	Learning parameter within the same rater in repeated blocks	Character stability (learning rate—like measure) between repeated blocks of the same rater, *λrep*	Mathematically similar to *λgen* presented above, but between repeated blocks of the same rater
9	Memory parameter	Memory (decay rate) parameter determining forgetting of evidence from one trial to the next, *mem*	A higher *mem* leads to less forgetting from one trial to the next
Rater response function			
10	Weight of attribution to the rater’s positivity policy	Weight of Attribution in Rater’s positivity policy function, *wAttr.* Relevant in ‘refine’ learning	A higher *wAttr* leads to a steeper change of policy with attribution (i.e., the slope of the sigmoid of attribution). A very high value would correspond to a step-like ‘switch’ where very low or very high probabilities of giving positive feedback are obtained, as the underlying trait (i.e., the attribution) changes smoothly. In the winning model, this was fixed to a single value across participants
11	Bias	Bias (intercept) in Rater’s positivity policy function, *w0*	A higher *w0* denotes an overall bias towards positive feedback for each rater. Fixed to a single value across participants in the winning model

We then considered learning and forgetting parameters, and found that both ‘*classify-refine*’ and ‘*beta-belief*’ models benefitted from their inclusion as a parameter group (model parameters 7 to 9 in Table 4), consistent with previous work^14^. Similarly, both classes of model benefited from the inclusion of a Rater response function, as per^16^. We then explored the balance of model accuracy and parsimony as follows (summarized in Fig. 4).

The fit of ‘*classify-refine*’ models improved by keeping distinct parameters for Attribution and Policy Certainties (model parameters 3 and 4 in Table 4) but merging the self-other dimension in Confidence. A noise floor or lapse parameter improved the fit, but there was no evidence for right-left lapses (model parameter 6 in Table 4; also see Supplementary Information). Somewhat to our surprise, all three learning and forgetting parameters were beneficial. Finally, model fit improved if the rater response parameters were fixed across the whole sample.

‘*Beta-belief*’ models were improved by adopting a simpler, population-wide rater response function that did not contain an attribution weight (model parameters 10 in Table 4) and had a fixed bias parameter.

We tested the ability of the winning ‘*classify-refine*’ models to capture data features by dividing each block into early, middle, and late trials, calculating weighted averages of each segment, and testing whether experimental data and synthetic data correlated for each of these. We obtained highly significant correlations, which were modest in value, and unsurprisingly, given that there were effectively less than 7 trials per segment (see Fig. S4).

#### Mental health symptom findings

We then performed an exploratory multivariate linear regression to investigate whether anxiety symptoms can be predicted by parameter(s) of the winning ‘*classify-refine*’ model, with the dependent variable as the ‘Anxiety’ factor score, and independent variables as the best-fit parameters from the winning ‘*classify-refine*’ model (orange framed in Fig. 4). The regression model was significant, *p* = 0.047, adjusted R-squared = 0.064. The significant *β* weights were those for policy certainty *aEv*, *β* = 0.23, *p* = 0.028, and decision noise *αPrec*, *β* = -0.29, *p* = 0.018. This means that people reporting higher levels of internalizing symptoms showed a greater decision noise. In addition, a reduced propensity to refine their attributions indicated stronger priors about the policies of other people. Altogether, these findings are consistent with greater decision noise often increasing with symptoms in a variety of tasks in the literature. However, regression of all task parameters against our GAD-7 Anxiety questionnaire score was not statistically significant, *p* = 0.47.

As around one-third (33.8%) of the participants reported a current or historic diagnosis of anxiety or depression (see Table S3, Table S5, and Fig. S2), we explored whether this was statistically explained by winning model parameters. Multiple logistic regression analysis with self-reported mood disorder diagnosis as the dependent variable, and independent variables the values of the best-fit parameters from the ‘*classify-refine*’ model were significant, *p* = 0.017, McFadden’s R-squared = 0.11. Only decision noise *αPrec* exhibited a significant effect on mood disorder diagnosis (*β* = − 0.83, *p* = 0.011). This means that decision noise explains self-reported mood disorder diagnosis, comparably to ‘Anxiety’ factor scores, but policy certainty did not *(p* = 0.97).

We then tested whether symptom factor scores explained self-reported mood diagnosis. A logistic regression with ‘Anxiety’ factor scores as independent variable, and self-reported mood diagnosis as dependent variable showed that the ‘Anxiety’ factor scores predicted self-reported mood diagnosis (*β* = 0.7593, *p* = 1.48e-4). This suggested that current symptoms explain a large variance (76%) in vulnerability to clinical mood disorder.

Finally, we examined whether symptom factor scores mediated the predictive effect of decision noise on mood diagnosis. Logistic regression with Anxiety factor scores and decision noise as independent variables showed that the effect of decision noise survived correction for ‘Anxiety’ factor scores, (*β* = − 0.63, *p* = 0.041), with the latter, as might be expected, also being highly significant (*β* = 0.69, *p* = 6.64e−4). Explanations for the difference between clinical diagnoses and symptoms might be the use of dichotomous (diagnosis) versus continuous (symptom) scales, or between clinician versus self-report rating. Thus, model parameters usefully explain variance in the likelihood of diagnosis of a clinical mood disorder over and above current symptoms, but further research is needed to elucidate its precise nature.

#### Parameter recovery

We performed recovery parameter studies after deciding on the winning model. This indicated strong recovery for all the parameters involved in our hypotheses (See Supplement Fig. S7A to S7I for details.

## Discussion

We sought to deepen our understanding of the computational mechanisms of social evaluation, a key construct in internalizing conditions such as depression and anxiety. We developed an existing cognitive task, the SELT, to improve its ecological validity, especially for use in younger populations. Stakeholders’ views expressed in focus groups improved the language, framing, and tolerability of the task, particularly for children and young people. We introduced changes to the delivery of the SELT-R to provide more data per trial and per participant (e.g., use continuous ratings instead of binary ratings) and proposed new computational models to better understand the rapid inference found in this task. Our new ‘*classify-refine*’ models, which allowed participants to ‘jump to attributions’ and then refine them, outperformed our previously published inference (‘*beta-belief*’) models^14,17^.

Focus group feedback led to several improvements in the task. Young people found the original task too long and lacking a clear objective. In response, we improved the task framing to be about meeting new classmates, and a final decision to sit next to a classmate. We also adjusted the word pairs used to more developmentally appropriate and differentiated language. Participants liked personalizing their avatars and felt that it immersed them in the task. Final feedback suggested that the task was emotionally salient and socially relevant.

We are interested to render the task even more ecologically valid in the future. Some focus-group participants misunderstood aspects of the task, thinking that ratings changed based on their responses or reflected a personality test, highlighting the need for clearer instructions. Another important consideration is the further customization of chosen avatar characteristics and their impact on expected ratings. For example, whether expectations of disapproving feedback might relate to the ethnicity of the participant—avatar. Future versions of the task will 1) examine the effect of rater’s and ratee’s ethnicity and gender on response, and 2) collect qualitative data on participants’ understanding of their perceptions and responses.

The finding that ‘*classify-refine*’ models give a better account of behavior in this task aligns with recent research^18^ and provides support for the validity of the ‘*classify-refine*’ model as a framework for understanding how people infer attributes of others. These results indicate that the cognitive mechanisms postulated here are likely to be generalizable across experimental designs. Evaluating oneself and others is a universal experience, but differences in this evaluation process are central to many psychiatric disorders like depression and anxiety. Given the overlapping social-cognitive mechanisms across these internalizing disorders, understanding social learning as a transdiagnostic mechanism is pivotal. Non-computational models of polarized thinking are well-established in the psychological literature^8,19^. Individuals can develop extreme or polarized beliefs about themselves and others. This can lead to idealizing others by attributing exceptionally positive qualities to them that do not align with reality, or devaluing others by perceiving them as having unrealistically negative qualities^8^. Such polarized views of oneself or others are characteristic of several mental health conditions and personality dispositions. This lends some credence to our computational model. Moreover, the ‘classify-refine’ model worked well in a completely different attribution task, in which individuals made attributions about a computer partner who shared money with them (or not)^18^ However, whether such social inferences are generalizable to the current task setting, and be explained by the ‘classify-refine’ model this needs further research.

To test our hypothesis that individuals with higher internalizing symptoms scores would show different social learning mechanisms, we first optimized each class of model (i.e., ‘*classify-refine*’ and ‘*beta-belief*’), so that improvements made in one class were also explored in the other class, to ensure a fair comparison. This led to an interesting finding, namely that both classes of models benefitted from a richer response function incorporated in the generative model, and from fixing the respective response functions across the population. In psychological terms, this aligns with an acknowledgment that individuals are involved in intricate socio-cognitive processes, a phenomenon that is worth consideration. However, it emphasizes the importance of limiting additional complexity, particularly that unrelated to our central hypothesis. This is the modeling equivalent of reducing the number of exploratory comparisons in frequentist statistics, which include all variables indiscriminately in explaining behaviors.

We found significant relationships between task parameters and current symptoms, as well as lifetime diagnosis of mood and anxiety disorder. Our measure of decision noise correlated with an ‘Anxiety’ factor in the direction expected from previous work, i.e., higher symptoms associated with greater decision noise^32^. We also found evidence that the ‘refine’ process was less flexible in those with higher symptoms, i.e., they had stronger priors about what a ‘positive’ or ‘negative’ rater would do. Model fit reduced as ‘Anxiety’ factor scores increased, suggesting that a high decision noise may be due to models imperfectly capturing how people with higher anxiety symptoms think and feel. We found no evidence that performance on the revised task was statistically related to self-reported levels of social anxiety.

Decision noise, but not policy decision, also emerged as a significant predictor of mood disorder diagnosis, and this effect survived correction for Anxiety factor scores, which also robustly (but imperfectly) predicted lifetime diagnosis. This aligns with clinical research emphasizing the low stability of mood disorder diagnosis across lifetime^33^, so that current symptoms are distinct from past diagnoses, symptom scores containing both trait and state information. Our findings in this domain are exploratory and in need of replication in future research, but they still suggest interesting mechanistic hypotheses—namely, that decision noise is more of a trait characteristic and is useful in predicting lifetime clinical need over and above current anxiety measures, but that the attributional rigidity captured by the policy confidence parameter may just be part of state-like anxiety states.

Our research on social learning in internalizing disorders uncovers subtle differences in social learning mechanisms by refining the SELT task and identifying the superiority of the '*classify-refine*' over ‘*beta-belief*’ model. These findings offer insights into transdiagnostic social-cognitive mechanisms and potential mechanistic hypotheses for mood and anxiety disorders.

Our study has several limitations. Firstly, the sample size was relatively small (*n* = 130) and contained modest variation in symptomatology, resulting in limited reproducibility and generalizability of our findings. Moreover, there is the limited scope of clinical measurements used here, resulting in robust factor scores only about anxiety. Furthermore, the incorporation of attentional checks might be systematically confounded by careless and/or insufficient effort that might be clinically meaningful, despite the original aim of filtering out inattentive individuals^34^. Future studies should incorporate a broader range of measures, including measures of confidence, prosociality, and conditions traditionally associated with cognitive rigidity, especially obsessionality, schizotypy, and eating disorders. This would help capture a comprehensive picture of ‘anxious’ and ‘depressive’ people—evaluations across dimensions of psychopathology. Finally, as our exploratory study revealed an unexpected main effect of ethnicity, in which a positive discrimination was found in non-White compared to White ratees. We intend to further investigate its impact on social learning in forthcoming research.

## Conclusions

This study enhances our understanding of the computational mechanisms in social learning, focusing on self and other social evaluations linked to anxiety and depression. The development of the SELT-R with focus group input provides an engaging tool to study social learning in young people. Findings support '*classify-refine*' mechanisms to explain social evaluation, and decision noise in predicting internalizing symptoms and mood disorder diagnosis. This research lays the groundwork for further exploration into cognitive mechanisms in internalizing disorders, particularly in young people, and towards a broader understanding of social learning processes in psychiatric conditions.

## Methods

### Participants

Online recruitment was conducted on Prolific^23^. The study was pre-registered (see https://osf.io/s25dn). The University College London Research Ethics Committee approved the study (Ethics Project ID number: 22135/003), and all participants provided informed consent. This study was conducted in accordance with the Declaration of Helsinki. Inclusion criteria were: (1) the absence of any self-reported history of severe neuropsychiatric conditions (e.g., severe learning disabilities, schizophrenia, or post-traumatic stress disorder), (2) aged between 18 years to 65, (3) currently living in the UK, (4) fluent in English, and (5) the absence of uncorrected visual impairments. Based on a mixed-model design, a sample size of 100 participants was determined to provide 80% statistical power for detecting an effect size (d) of 0.57, using a 5% significance level^35,36^. To ensure an adequate sample size and account for potential dropouts and quality control, initial recruitment included 154 participants, who all gave informed consent. However, 24 participants were excluded due to failing both attentional checks (*n* = 6) and technical issues within the Prolific platform and the model-fitting scripts (*n* = 18). The final sample comprised 130 participants.

### Measures

The SELT-R is a modified version of the original SELT^10^, which aimed to investigate social evaluation learning in individuals. In this task, participants were required to accumulate knowledge about whether a computerized persona (in either a self-referential or other-referential context) was liked or disliked by another persona. This learning process was facilitated through feedback provided as pairings of positive and negative personality words (e.g., caring/uncaring). SELT-R consisted of eight distinct blocks: self-liked, self-liked-repeated, other-liked, other-liked-repeated, etc., (see Introduction). At the beginning of the block, the task introduced the rater (which was always new except in the case of the repeat blocks) and indicated whether the self-character or the other character was being rated. The character was always the same in the other-referential blocks. Each block comprised 20 trials. Participants rated the probability (0 to 100) of the rater selecting the positive word in each trial. The correct word was circled based on block rules (i.e., liked, neutral, or disliked) after participants rated. At the end of each block, participants provided a global rating stating how often they thought the rater described them positively (self-referential) or the other computerized persona (other-referential). No feedback was presented after the global ratings.

Compared to the original SELT^10^, the SELT-R introduced several enhancements. It introduced two new blocks, "self-liked-repeated" and "other-liked-repeated." The sequencing of these blocks, originally randomized, was carefully pseudo-randomized in the SELT-R to ensure that "liked-repeated" blocks (e.g., a repeated block with the same rater as the preceding "dislike" block but with the feedback contingency aligned with the "liked" rule) consistently followed "disliked" blocks of the same referential condition. For example, the self-referential “disliked” block must be followed by the self-referential “liked-repeated” block. This enabled us to mitigate ceiling effects within “liked” blocks, which were primarily attributed to a positive bias (i.e.,the presumption that individuals are liked). Participants tend to initially expect positive feedback—which creates a ceiling effect for learning from positive feedback. The repeat block system aimed to mitigate this ceiling effect in the "liked" blocks by first establishing an expectation of being disliked by the rater in the "disliked" block. This lowered participants' initial positive expectations for the subsequent "liked-repeat" block. By reducing this positive bias, the system allowed for better capturing how participants updated their beliefs based on positive feedback, removing constraints on their potential for learning from positive information at the start of each block. Moreover, based on feedback from focus group sessions, participants could personalize their avatars, fostering a sense of identification with their virtual persona. Additionally, a cover story was integrated, framing the task within a school environment at the beginning of a new academic year. Participants were tasked with selecting “classmates” (i.e., raters) they would prefer to sit next to, further emphasizing the social context of the task.

Following preliminary studies and the limited recovery of the initial bias parameter in previous studies^14,17^, SELT-R included eight blocks, a notable expansion from the original version. It also employed continuous measures (e.g., “20% likely to be dull,” “80% likely to be exciting”) rather than binary measures, aligning better with the nuanced nature of human decision-making and providing more bits of information per trial. Two attentional checks (i.e., where participants were asked only to simply move the slider to 100) were incorporated to ensure participants' engagement and focus throughout the task. The attention checks were failed if instead participants randomly provided a value between 0 and 100, indicating they had not read the instructions. This effectively excludes inattentive responses for a more robust assessment of social learning..

In addition to the SELT-R, participants completed questionnaires of internalizing symptoms assessing anxiety and depression, which included GAD-7, PHQ-8, STAI-Trait, and SMSAD-A.

### Focus group

We ran two focus groups with young people to get feedback on several elements of a wider study looking at emotion processing and mental health in young people. Given the groups’ advisory function, we obtained consent from participants and parents for sharing findings from the focus groups, but not sharing transcripts or direct quotations. We did not collect demographic data from participants. All participants were compensated for their time.

The first focus group was recruited from and held at a private girl’s school in London. The participants were 11 girls aged 14–16 years. In this session, we showed participants screenshots from the SELT task in development and explained the flow. Discussion of this task lasted 20 min. The second focus group was recruited from and held at a community organization in London. The participants were 10 young people, mixed in gender and age, recruited within the range of 13–18 years old. In this session, participants played the task in full on their mobile phones or laptops and then discussed it. For 20 min, some feedback was given while participants played the game, and then there were 15 min of discussion.

In both sessions, we asked questions to probe participants’ understanding of the task and their approach to completing it, as well as their general opinion and experience of it. We were also interested in several specific features of the task, e.g. whether any of the words in the word pairs were noted as too complex, what they thought about the option to have an avatar, and whether they would be able to remember the repeated characters. The discussion was loosely guided by these key questions, but some key findings were spontaneously contributed by participants, e.g. both groups were interested in how ethnicity/skin tone and other visible characteristics of ‘raters’ and ‘ratees’ might affect ratings and/or participant answers.

### Procedures

Participants began the study with the SELT-R task, followed by another associative learning task unrelated to this study. The battery of self-report questionnaires was then administered to assess symptoms of anxiety and depression. The entire study session lasted for approximately 30 min, and participants received reimbursement proportional to £9 per hour.

### Factor analysis

An exploratory factor analysis (EFA) was conducted on the combined items from each symptom questionnaire. The analysis employed orthogonal rotation (varimax) to unveil the inherent structure of the items. Prior to conducting the EFA, parallel analysis and scree plot were carried out to establish the most suitable number of factors (see Fig. S3).

### Model fitting

The process of fitting models involved implementing maximum-a-posteriori (MAP) fitting with weakly informative priors, as defined over the native parameter space^37–39^. Subsequently, the sum-log-likelihood at the MAP estimate was utilized to compute BIC values for each participant. In instances where a model parameter was estimated across the entire participant dataset (e.g., the parameter values were constrained to a single value for all participants), we adjusted the complexity penalty associated with that parameter to align with the variants of BIC, as an approximation to model evidence^40^ (see Eq. S11). This allowed us to include an appropriate complexity penalty for mechanisms that we felt might be important to describe cognition overall but did not relate to our study hypotheses and hence should not be allowed to compete to explain variance^40^.

In the course of model-fitting, we found that gradient-descent methods had considerable problems with local minima. Hence, we resorted to a custom, iterated, adaptive grid search optimization. It involved repeatedly calculating the log-likelihood across a grid of parameter values, with each parameter adjusted individually at a time. Initially, the grid spanned the entire psychological range of interest for each parameter. For instance, if a parameter's range extended from 0 to 1, we initially utilized a coarse grid spanning this full range (e.g., a 12-point grid from 0.05 to 0.95). However, recognizing the limitations of this coarse grid, we refined it in subsequent iterations. In these refinements, the grid was centered at the best-performing parameter value identified in the previous iteration and was narrowed to cover a smaller range, thus increasing its resolution.

### Regression analyses

The utilization of weak fitting priors within the native parameter space primarily led to parameter distributions in the transformed space that resembled normal distributions, where subsequent regression analyses were conducted. However, this approach also allowed for the presence of outliers within this transformed space. Consequently, we employed the linear and logistic regressions in R^41^. The model used is as follows: model <—glm(Anxiety ~ pS + pO + dEv + aEv + αPrec + λgen + λrep + wp0 + wAttr + mem + lps, family = gaussian). For exploratory logistic regression model: model <- glm(Anxiety ~ pS + pO + dEv + aEv + αPrec + λgen + λrep + wp0 + wAttr + mem + lps, family = binomial).

### Ethical approval

In accordance with the Declaration of Helsinki and guidelines and regulations governing research involving human participants, we confirm that all methods employed in this study were conducted in compliance with the relevant ethical standards. The University College London Research Ethics Committee granted approval for this research (Ethics Project ID number: 22135/003), and all participants provided informed consent.

### Supplementary Information

Supplementary Information.

## Data Availability

The data supporting the findings of this study are openly available in the Open Science Framework (OSF) repository (https://osf.io/zwspj/). The repository includes all relevant scripts and datasets. Access to this data will be provided upon manuscript acceptance. For inquiries regarding the data or additional information, please contact the corresponding author.
