# Supplementary material for: Jumping to attributions during social evaluation
Source: Sci Rep. 2024 Jul 4;14:15447. doi: 10.1038/s41598-024-65704-y (PMC11224235; doi:10.1038/s41598-024-65704-y)
Supplement: Supplementary file 1 — Supplementary Information. [file 41598_2024_65704_MOESM1_ESM.docx]

Supplementary Information for:

Jumping to Conclusions during Social Evaluation

Isabel H.W. Lau, Jessica Norman^^^, Melanie Stothard^^^, Christina O. Carlisi^$^ and Michael Moutoussis^$^

^ These authors contributed equally

$ Joint Supervising Authors

# Supplementary Methods: Details of Computational Modeling

## 1.1 Classify-Refine Models

Classify-refine models were based on a 'Core' Hidden Markov model, formulated using the Active Inference suite in SPM. As such, they were characterized by beliefs about states of the world, here, the attributes of the rater, beliefs denoted by *d.* In a simplification of the active-inference frame, these belief states were mapped to participant actions through a parametrized yet fixed map (actions were passively inferred, so to speak). The attribute states were mapped to the likelihood of positive or negative word outcomes, by a map *a*. The elements of *d* and *a* were concentration parameters that quantified the strength of belief. As we shall see, they were subject to update via the credit assignment and subsequent learning formulation of Active Inference. We now turn to the component stages of these models in turn (see also **Fig. 2** in the main text).

**1.1.i Beliefs about Raters being positive or negative**

Agents were formulated to hold a belief that the Rater was either 'positive' or 'negative'. In addition, they held (uncertain) beliefs about what ‘positive’ and ‘negative’ meant, in terms of exact policy. For example, they could be 60% certain that a rater is positive, and 40% that they are negative, but also that a positive rater gives 80% positive feedback and 20% negative feedback (i.e., their policy likelihood).

Beliefs about raters were parameterized by notional counts, or concentration parameters *d_+_* , *d _--_* . Their initial values for the whole experiment were set through the model parameters as follows:


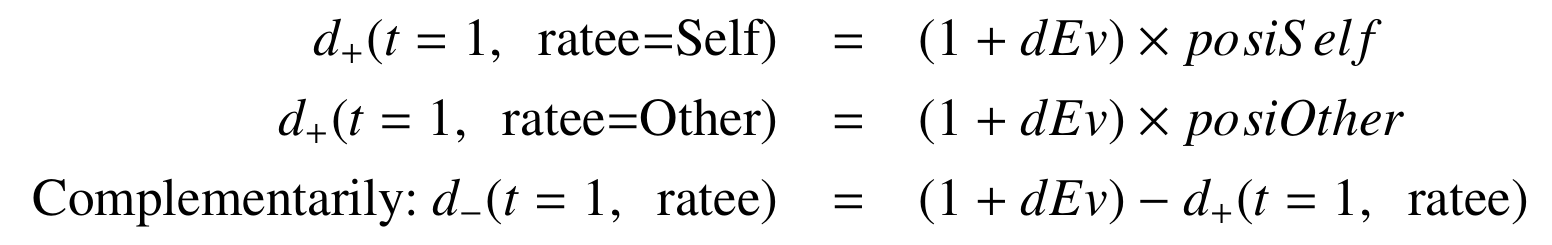


*Eq. S1*

At the end of a trial, the posterior belief that this particular rater was positive vs. negative was accumulated in *d* to form the new prior. The additive 1 in (1+*dEv*) rendered parameter fitting of *dEv* better behaved.

*d* thus contained 'negative' (*d_-_*) and 'positive' (*d_+_*) states which, within the active inference frame were just categories. To map these to numerical attributes that raters would choose the positive word, and to visual analogue scale (VAS) responses, we corresponded the 'negative' and 'positive' states to attributes*,* *A_low_* and *A_high_* , intially 0.05 and 0.95 but, as we shall see, subject to learning.

At the same time, however, only a proportion *mem* of *d* (effective counts of the times the state in question has been visited) was carried over to the next trial, modeling 'forgetting' of evidence.

**1.1.ii Response function:**

In our simple Partially Observable Markov Decision Process (POMDP), the mean and confidence re. the attributes encoded by *d*, were used to form a beta-distribution over the VAS used for reporting. We then discretized *A* as well as the experimental data over a discrete, equally spaced, 6-bin Likert-like scale *A_j_, j=1...6* . *BetaCDF* below is the cumulative distribution, with concentration parameters *a_A_* (alpha [α]) and *b_A_*  (beta [β]):


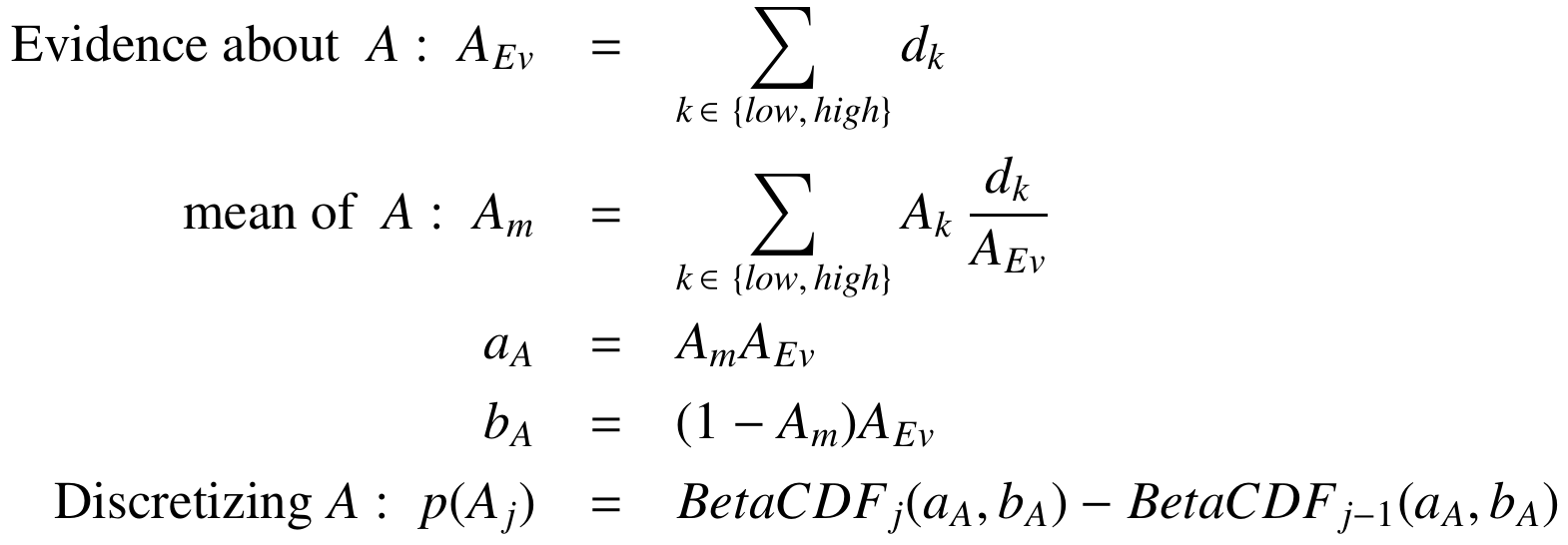


*Eq. S2*

We then tested three decision-noise models:

a. Softmax: Attributions were drawn from the belief vector (e.g., a vector containing probabilities for each of the 6 bins of attribution, in which we divided the VAS responses that the participants provided), sharpened by the policy-precision (inverse decision-noise) parameter *αPrec* :


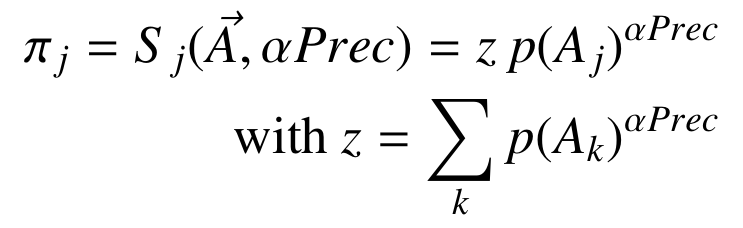


*Eq. S3*

Here, the Softmax function is :


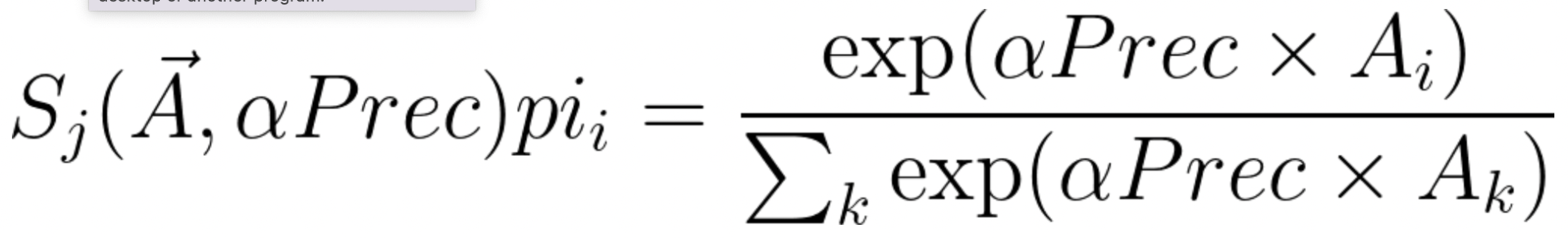
  *Eq. S4*

b. Softmax with uniform lapse process: This turned out to be the winning model. It is the standard uniform noise with the level of background noise parameter *lps* used in reinforcement learning, here for *K =* 6 (e.g., corresponding to the 6 bins) options:


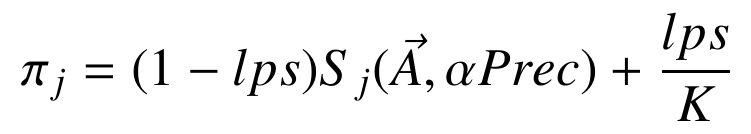


*Eq. S5*

b. Softmax with right-left lapse process: In our task, which end of the VAS corresponded to the positive word was randomized across trials, to reduce response bias. We checked whether this caused lapses in the sense of the participant forming an attribution of positivity, but confusing which end of the scale corresponded to the positive word:


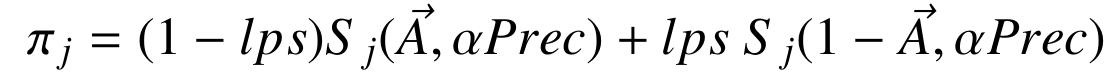


*Eq. S6*

**1.1.iii Likelihood matrix: What does a 'positive' rater do?**

In our classify-refine models, the *A (attribution)* did not equate to the expectations regarding observations. For example, a participant might feel sure/ attribute that a rater was 'positive' and so report *A_high_* = 95%, but their (latent) expectation might be, say, 80% because of two processes, which we modeled. The first was a positivity bias *w0*, accounting for social desirability to make benevolent attribution about raters. The second was an attribute coefficient *wAttr*, accounting for distortion in probability reporting. We combined these to define the likelihood matrix *a* of active inference, which gives the belief that an observation will be made given an underlying state:


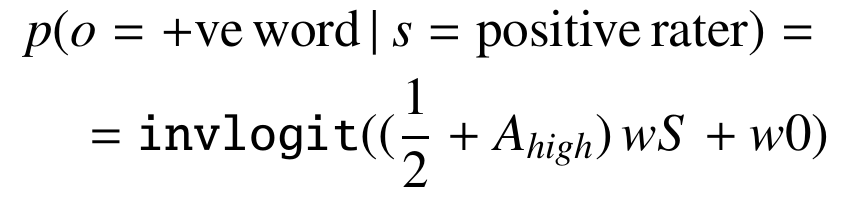


*Eq. S7*

and similarly, for the other three combinations of state and observation. These probabilities were converted to initial values of the entries of the *a* matrix, which are not probabilities but effective counts in the exact same manner as per Eq. S1, but now using parameter *aEvInit* rather than *dEvInit*.

Crucially, from trial to trial the concentration parameters of *a* were subject to learning, which constituted the 'refine' element of the classify-refine models. This followed the standard active-inference scheme [(1)](https://www.zotero.org/google-docs/?6sIY39), with *mem* being the same information-retention / 'forgetting' parameter as for beliefs about states (*d*):


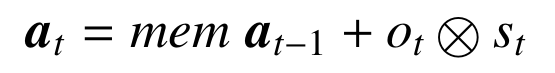


*Eq. S8*

## 1.2 Beta-belief Models

These were close to our published Bayesian models [(2)](https://www.zotero.org/google-docs/?R44hcK). In the light of further work [(3)](https://www.zotero.org/google-docs/?EEobyA), they were simplified with respect to the self- and other-baseline representation. As before, participants were described as holding beta-shaped beliefs about themselves and others, characterized by positive and negative (notional) evidence about self and other, *α_Self_*, *β_Self_*, *α_Other_*, *β_Other_* .

The initial values of *α_Self_*, *β_Self_*, *α_Other_*, *β_Other_* were set exactly like *d_+_*(*t =* 1, ratee=Self),

*d_+_*(*t =* 1, ratee=Other), etc. respectively in Eq. S1.

These were then subject to evidence accumulation and forgetting, again subject to a memory parameter *mem* :


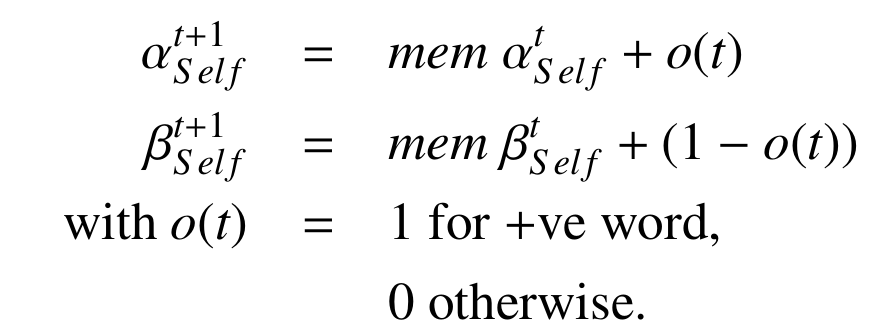


*Eq. S9*

and similarly, for *α_Other_*, *β_Other_*.

The resulting beliefs gave rise to decision-making via a response function. We tested two response functions, the logistic-sigmoid form of Eq. S7 and a simpler one, which just used *w0* to bias expectations. Note, however, that in these (non-active-inference) models, this function subserved a different role. Rather than mapping attributions to actions of the rater in the generative model, it was placed between attributions and actions *of the participant.* Hence, although the parameters *wAttr* and *w0* performed in the end a somewhat comparable function, the structure of the model is different (i.e. same as the published one).

## 1.4 Between-block learning

The current task had 8 blocks, two of which were repeated-rater blocks. That is, the same rater-ratee pair was encountered again. Participants were told that the rater may be in a similar or different ‘mood’ the second time compared to the first. In effect, the repeated-rater blocks were instances of reversal learning, so that across participants the first repeated-rater block was with a 20% positive rater, and the second with an 80% rater. We hypothesized that hence, the second block would start with on-average reduced positivity bias, avoiding possible ceiling effects. However, this scheme meant that a simple method for modeling between-block learning was needed, and that this would be different between same-rater and different-rather blocks.

To do this, at each block we calculated the shift in beliefs from prior mean to posterior mean over the entire block. In this very simple approximation, we fitted a single learning-rate-parameter *λ* to account for belief shift and evidence accumulation, where *σ* is the standard deviation of a probability distribution:


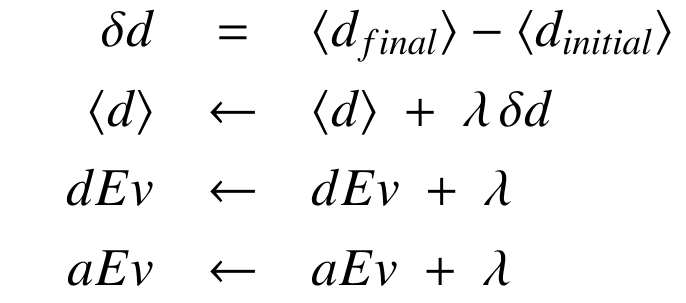


*Eq. S10*

We did, however, allow for two different learning parameters *λ_repeat_, λ_general_* to account for learning within (repeated) rater vs. across different raters, as these were very different kinds of learning. Eq. S9 illustrates learning between blocks for the Classify-refine model, but the Classic and beta-belief models followed exactly the same pattern, using the shift in mean beliefs about states over the block to update prior beliefs for the next block.

**1.5 Model-fitting measures and BIC approximations**

In this study, we used variants of the Bayesian Information Criterion (BIC) to optimize parameter fitting and provide goodness of fit measures. Fitting individuals using weakly informative prior distributions over parameters and maximum-a-posteriori fitting (MAP) ensured that the variance associated with conditions not included in the fit was preserved, for subsequent analyses. This is a practical method of model-fitting when the objective is to optimize power to explore correlations with psychometric variables [(4)](https://www.zotero.org/google-docs/?vgXzJK), although it comes at a cost of somewhat reduced accuracy in the estimation of parameter values (as opposed to their correlations with ‘external’ measures).

We then used the parameter values of the MAP estimate in BIC formulae for each individual. The BIC is an approximation to the Model Evidence, i.e. the probability that the observed data *D* was produced by a model *M.* Considering one participant, it is given as follows [(5)](https://www.zotero.org/google-docs/?IhS7Ve):


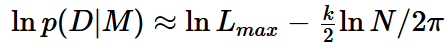
 *Eq. S11*


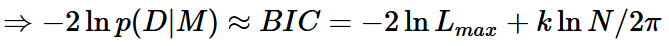


Here, k is the number of free parameters of the model (formally, degrees of freedom) and *N* the number of data points. The last term can be re-written as *k* (ln *N* - ln 2*π* ), and the last term of this is sometimes considered negligible.

However, in some of the models that we considered, not all parameters are freely fitted to each individual. Instead, we considered whether more parsimonious (and with better parameter recovery) models could be formed by using a single parameter value for all participants, and fitting the other parameters freely, as explained in the main text. If a number *c* of parameters is constrained, we cannot simply use k-c instead of k in Eq. S11, as we have already used the *N* datapoints to derive the *c* parameters - an additional complexity penalty is needed for this. To estimate this complexity penalty, we observe that the complexity penalty for parameters fitted over the entire population sampled is approximated by the complexity penalty term of the ‘integrated BIC’, which is [(6)](https://www.zotero.org/google-docs/?b7LSA6):


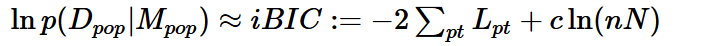
 *Eq. S12*

Where ‘pt’ indexes each participant, ‘pop’ denotes the population, *c* denotes the number of free parameters fitted to the entire population (e.g. population means, population SD of a parameter), *n* is the number of participants, and *N is* the number of data points per participant, as before. *L_pt_* is the log-mean-likelihood per participant, where the mean integrates over possible parameter values at the participant, rather than the population, level. It does not concern us here. We therefore observe that the average complexity penalty per participant for the population-wide parameters is *c/n* ln (*n N*). We therefore add this correction to arrive at


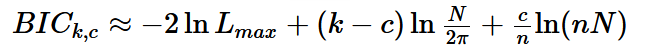
 *Eq. S13*

In the course of model-fitting, we found that gradient-descend methods had considerable problems with local minima. Hence, we used a custom, iterated, adaptive grid search optimization. To do this, we formed a grid of 12 points over each parameter, which initially covered the entire range of psychological interest. We then searched for optimal optimal values of each parameter sequentially. This partly guards against local minima, compared to gradient descent, in that it examines the whole parameter range, but it offers no absolute guarantee as it does not examine all possible parameter combinations (which are essentially infinite). We therefore used a further iteration, this time across models with nested parameter sets. We tested that first, more complex ones did not have a worse log-likelihood than simpler ones (this was uncommon but not rare). If so, the parameters of the simpler modes were used as initial values for the more complex grid. Second, we tested that the winning model did not win simply because others fell into local minima. To do this, we used the values of in-common parameters from the more complex model to initiate the less complex ones.

# Supplementary Results

## 2.1 Notes on minor technical issues

We initially recruited 154 participants from the UK online on Prolific [(7)](https://www.zotero.org/google-docs/?CVpFyr), and 24 were excluded—6 for failing attentional checks and 18 due to technical issues on the Prolific platform and task scripts. The final sample included 130 participants.

## 2.2 Focus group sessions results

| **Focus group feedback** | **Proposed change and rationale** | **Lessons learned for future modifications** |
| --- | --- | --- |
| - Task too long. - Task lacked overarching aim. | - Add back story of meeting new classmates to make the task relatable and engaging. | - Added overarching goal of choosing who to sit next to in class, in addition to just seeing what classmates think of you/the other character |
| - Several words were flagged by participants as being difficult to comprehend. | - Edit word pairs to be more developmentally appropriate - Removed words that were ambiguously positive/negative (e.g. “*emotional*”). | N/A |
| - Certain words were too similar or related in meaning (e.g. “*brave*”, and “*confident*”). | - Separate closely related words into different task blocks. | N/A |
| - Participants liked inclusion of an avatar and felt that it made them immersed and invested in the task. - Participants were interested in having more complex avatar customisation, potentially including other characteristics e.g. picking hobbies | - Include selection of avatar to represent the participant at the beginning of the task. | - That this was out of the scope of the current study, although an interesting avenue for the future. |
| - Participants were interested in how ethnicity and/or gender of the rater might influence responses. - One participant thought that they might have received negative feedback because they had chosen an avatar with black skin. | - The avatars for both raters and ratees had a variety of characteristics, including skin colour and gender. | - This is an important potential avenue for future research using this task. - Participants in the focus group were debriefed that the feedback was random, and did not relate to the chosen avatar. - It may be important to debrief carefully to young people to ensure that they do not feel victimized by the task, and to avoid reinforcing gender or ethnicity related stereotypes. |
| - Participants felt that the raters’ responses were quite harsh. | N/A | - It will be important to consider the potential negative emotional effects of the feedback, and to debrief participants appropriately. |
| - Participants reported being able to recall specific raters and their opinions of them, and reported remembering that some raters had changed their minds. | - Added repeat blocks for self and other, to improve potential for measuring positive learning. | N/A |
| - Some participants thought the task might change the feedback based on the participants’ answers. One participant reported thinking that it was a personality test. Generally participants learned the premise of the task quite quickly, though. | N/A | - It would be interesting to explore individual differences in task interpretation and approach, so future versions could collect qualitative data on this at the end. |

***Table S1.*** Full focus group findings, proposed changes and rationale.

**2.3 Word pairs**

| **Positive word** | **Negative word** |
| --- | --- |
| wise | naive |
| mature | childish |
| trustworthy | untrustworthy |
| diligent | thoughtless |
| caring | indifferent |
| polite | rude |
| generous | greedy |
| precise | clumsy |
| active | lazy |
| focused | distracted |
| friendly | hostile |
| relaxed | tense |
| confident | self-doubting |
| sociable | avoidant |
| versatile | inflexible |
| consistent | inconsistent |
| exceptional | mediocre |
| organised | disorganised |
| supportive | critical |
| fun | boring |
| trusting | suspicious |
| rational | unrealistic |
| smart | foolish |
| responsible | reckless |
| warm-hearted | uncaring |
| helpful | selfish |
| lively | inactive |
| tidy | messy |
| patient | impatient |
| flexible | strict |
| alert | inattentive |
| enthusiastic | unenthusiastic |
| peaceful | argumentative |
| approachable | unfriendly |
| pleasant | obnoxious |
| tactful | tactless |
| committed | uncommitted |
| imaginative | uninventive |
| brave | cowardly |
| encouraging | negative |
| sensible | immature |
| honest | dishonest |
| loyal | disloyal |
| thoughtful | inconsiderate |
| sympathetic | mean |
| accurate | sloppy |
| motivated | aimless |
| settled | restless |
| curious | close-minded |
| cooperative | unhelpful |
| easygoing | irritable |
| gentle | harsh |
| outgoing | unsociable |
| modest | smug |
| fair | bossy |
| logical | irrational |
| witty | dull |
| talented | ordinary |
| competent | incompetent |
| productive | unproductive |
| clever | silly |
| reliable | unreliable |
| sincere | fake |
| attentive | careless |
| considerate | self-absorbed |
| kind | cruel |
| neat | untidy |
| open-minded | stubborn |
| eager | passive |
| charming | disagreeable |
| laidback | aggressive |
| calm | angry |
| humble | arrogant |
| well-liked | unpopular |
| determined | indecisive |
| practical | impractical |
| efficient | inefficient |
| cheerful | gloomy |
| bold | timid |
| tolerant | intolerant |

***Table S2.*** *Positive and negative word pairs*

## 2.4 Demographic and Psychometric results

| ***Demographic Characteristic*** | ***Subcategory*** | ***Count (%)*** |
| --- | --- | --- |
| **Gender** | Female | 65 (*50%*) |
|  | Male | 65 (*50%*) |
| **Ethnicity** | Asian or Asian British | 5 (3.9%) |
|  | Black or Black British | 3 (*2.3%*) |
|  | Prefer not to answer | 2 (*1.5%*) |
|  | White | 120 (92.3%) |
| **Parents’ educational attainment of or over bachelor’s during adolescents** | No | 86 (66.2%) |
|  | Yes | 36 (27.7%) |
|  | Not sure | 8 (6.2%) |
| **Received free school meals** | No | 95 (73.1%) |
|  | Yes | 28 (21.5%) |
|  | Not sure | 7 (5.4%) |
| **Mood diagnoses** | No | 86 (66.2%) |
|  | Yes | 44 (33.8%) |
| **Other Psychiatric Diagnoses** | No | 112 (86.3%) |
|  | ADHD | 5 (3.8%) |
|  | ASD | 5 (3.8%) |
|  | OCD | 5 (3.8%) |
|  | Two comorbid diagnoses | 3 (2.3%) |
| **Psychiatric medicine prescribed** | No | 119 (91.5%) |
|  | Yes | 11 (8.5%) |

***Table S3.*** Descriptive statistics of demographic variables.

|  |  | ***Trial-based positive ratings*** | | ***Global ratings*** | | |
| --- | --- | --- | --- | --- | --- | --- |
| ***Referential***  ***condition*** | ***Rule*** | ***Mean^a^*** | ***SD*** | ***Mean*** | | ***SD*** |
| Other | Disliked | 41.3 | 27.0 | 22.3 | | 17.7 |
|  | Neutral | 55.7 | 25.0 | 47.1 | | 14.3 |
|  | Liked | 67.8 | 21.8 | 70.0 | | 15.9 |
|  | Liked-repeated | 66.2 | 21.9 | 63.3 | | 16.4 |
| Self | Disliked | 47.1 | 29.5 | 23.5 | | 22.0 |
|  | Neutral | 57.1 | 26.7 | 43.2 | | 17.0 |
|  | Liked | 69.8 | 21.6 | 69.0 | | 16.7 |
|  | Liked-repeated | 67.0 | 22.6 | 66.6 | | 18.6 |

***Table S4.*** Mean trial-based positive ratings and global rating scores for referential condition and rule. *Trial-based positive ratings* are probability scores provided by participants before a trial-based feedback on whether they think the rater would select a positive word. A higher score reflects that participants believed it was more likely that the rater would provide positive feedback, and a lower score (e.g., closer to 0) indicated that participants believed that it was more likely that the rater would provide negative feedback in that specific trial. *Global ratings* are a summary scores indicating how much participants thought the rater liked them [self-referential] or another computerised persona [other-referential], on a scale ranging from 0 to 100.

|  | ***STAI-Trait*** | |  | ***GAD-7*** | |  | ***PHQ-8*** | |  | ***SMSAD-A*** | |  |
| --- | --- | --- | --- | --- | --- | --- | --- | --- | --- | --- | --- | --- |
| ***Gender*** | ***Mean*** | ***SD*** | ***p*** | ***Mean*** | ***SD*** | ***p*** | ***Mean*** | ***SD*** | ***p*** | ***Mean*** | ***SD*** | ***p*** |
| Female | 43.3 | 5.2 | <.001 | 6.7 | 5.5 | <.001 | 7.6 | 5.9 | <.001 | 9.2 | 9.6 | <.001 |
| Male | 43.0 | 5.3 | <.001 | 6.3 | 5.5 | <.001 | 6.4 | 5.6 | <.001 | 7.4 | 8.9 | <.001 |
| **Averaged** | 43.2 | 5.2 |  | 6.5 | 5.5 |  | 7.0 | 5.8 |  | 8.3 | 9.3 |  |

***Table S5.*** Descriptive statistics of psychometric questionnaires.


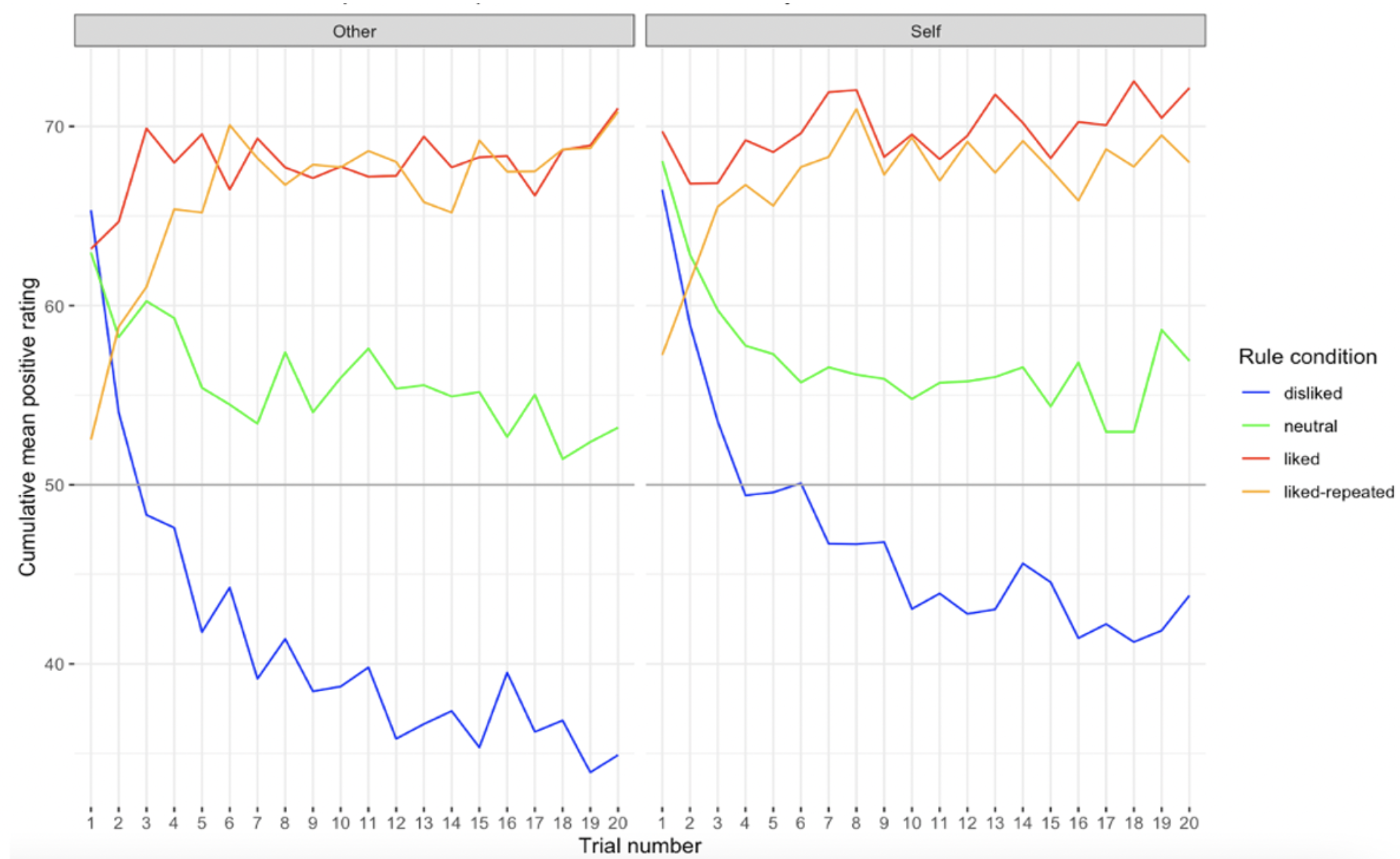


***Fig. S1.*** Cumulation of mean positive responses across the 20 trials conducted during the learning phase. The cumulation of mean positive responses across the 20 trials conducted during the learning phase was split by referential (e.g., self, other) and feedback rule (e.g., disliked, neutral, liked, liked-repeated). The distinct separation of curves by feedback rule indicated that participants adapted their responses accordingly.


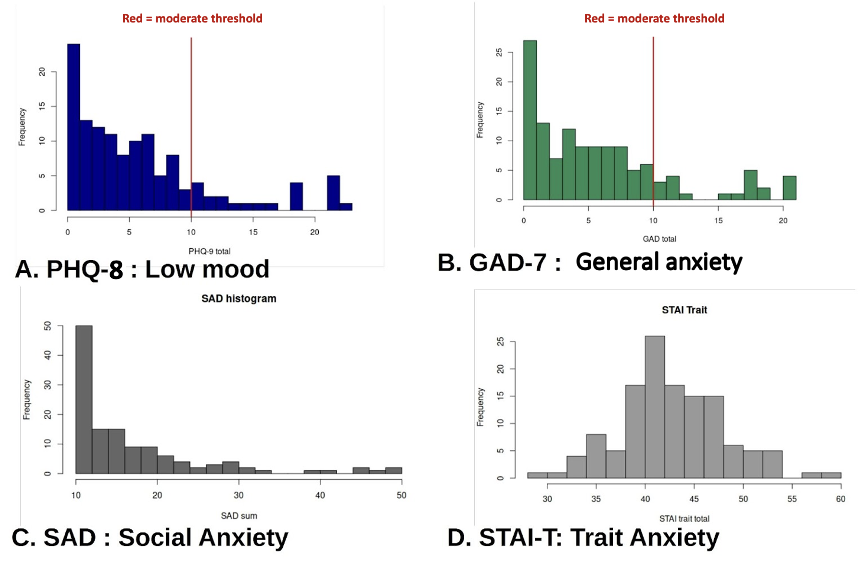


***Fig. S2.*** Histograms of symptom scores. Depression (**A.**), General Anxiety (**B.**), Social Anxiety (**C.**) and Trait Anxiety (**D.**) measures. Red lines in **A.** and **B.** are the conventional cut-offs between mild and moderate depression and anxiety respectively. As expected for a community sample, the most frequent symptom value is zero, and most participants have very low state symptoms. Again as might be expected, the Trait Anxiety distribution is much less skewed.


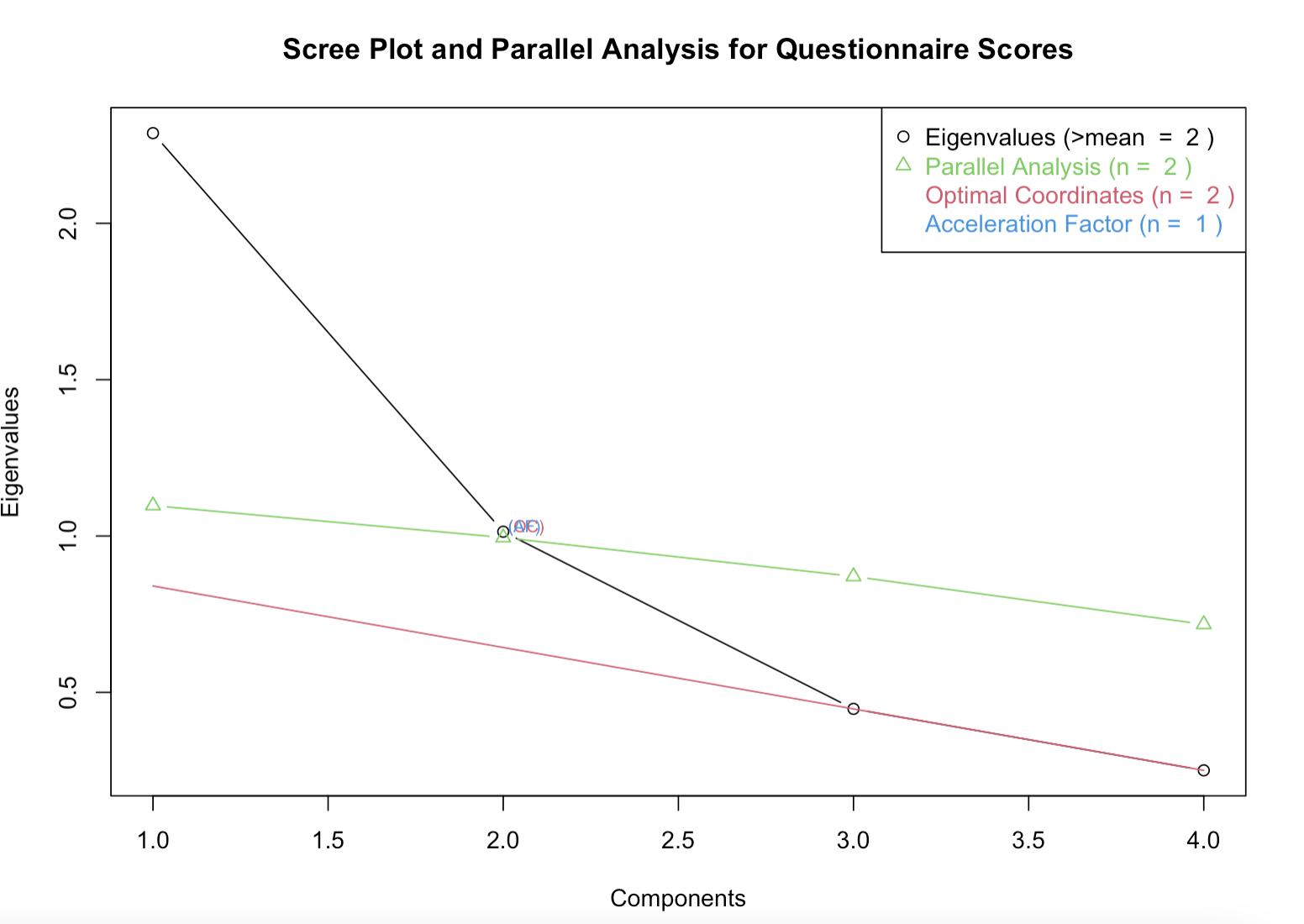


***Fig. S3.*** Factor structure analysis. Scree plot and parallel analysis results indicate the need for 2 factors to explain variance in factor analysis using overall scores for PHQ-8, GAD-7, SMSAD-A, and STAI-Trait.

| **Questionnaire** | **“Anxiety”** | **“Depression”** | **R²** | **Unique variance** | **Commonality** |
| --- | --- | --- | --- | --- | --- |
| STAI-Trait | .73 | -.21 | .58 | .42 | 1.2 |
| SMSAD-A | .79 | .17 | .66 | .34 | 1.1 |
| GAD-7 | .91 | -.05 | .83 | .17 | 1.0 |
| PHQ-8 | -.01 | .33 | .11 | .89 | 1.0 |

***Table S6.*** Factor loadings, R², unique variance, and commonality of the questionnaire scores on the two-factor structure. GAD-7: Generalized Anxiety Disorder 7, PHQ-8: Patient Health Questionnaire 8, STAI-Trait: State-Trait Anxiety Inventory - Trait version, SMSAD-A: Social Anxiety and Depression Scale for Adolescents.

|  | **STAI-Trait** | **SMSAD-A** | **PHQ-8** | **GAD-7** |
| --- | --- | --- | --- | --- |
| STAI-Trait |  |  |  |  |
| SMSAD-A | .13* |  |  |  |
| GAD-7 | .09 | .72** |  |  |
| PHQ-8 | .21* | .73** | .85** |  |

**Table S7**. Correlation matrix between the questionnaire scores. *: *p <* .05, **: p < .001. GAD-7: Generalized Anxiety Disorder 7, PHQ-8: Patient Health Questionnaire 8, STAI-Trait: State-Trait Anxiety Inventory - Trait version, SMSAD-A: Social Anxiety and Depression Scale for Adolescents.

##

## 2.5 Modeling results


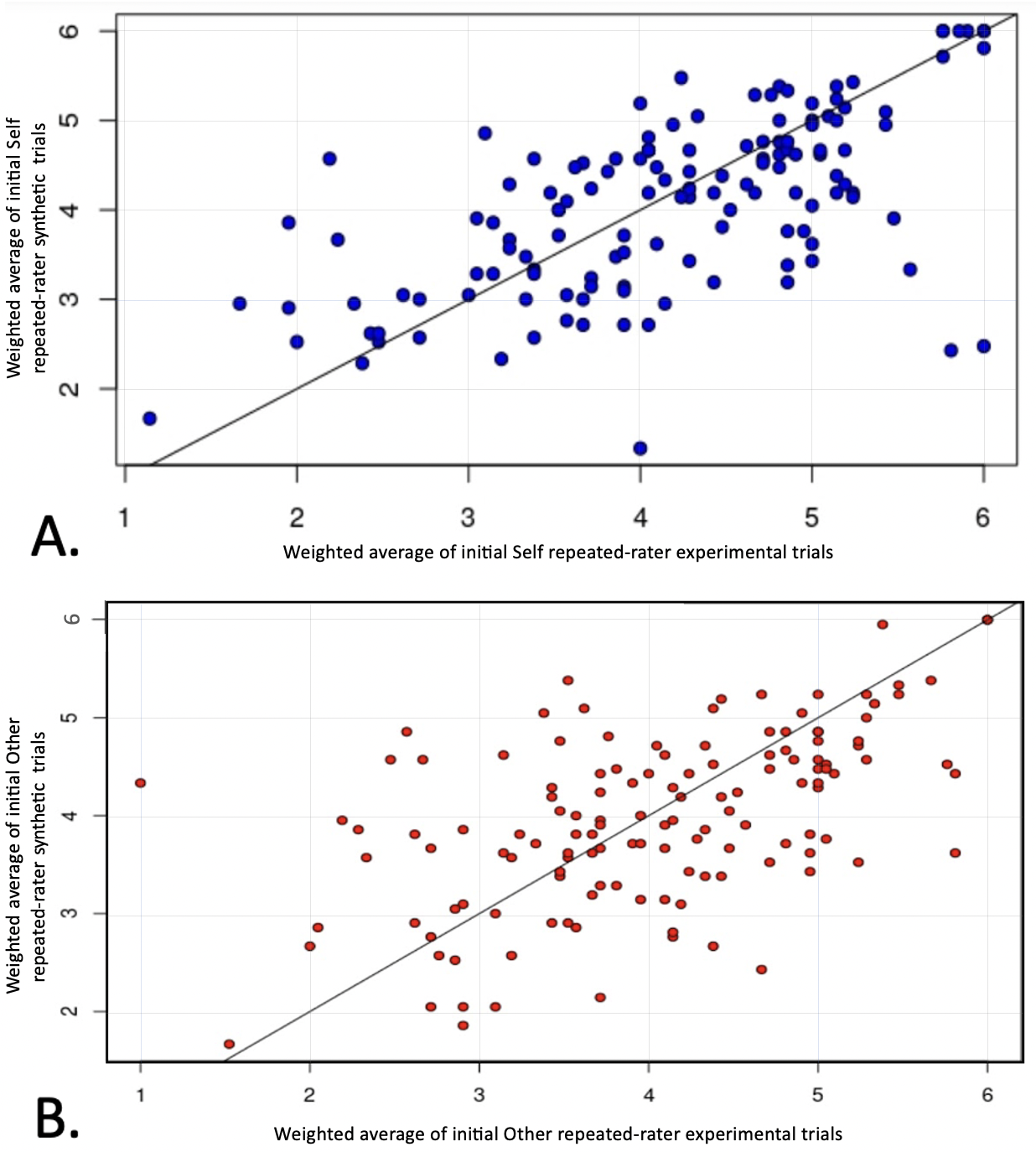


***Fig. S4.*** Correlation of key synthetic and experimental data. **A.** Weighted average of the first third of trials from the repeated-rater, ratee = Self block of experimental data (*abscissa*) vs. equivalent from synthetic data produced by the classify-refine model (in fact, model 8b in **Fig. 4**). *r =* 0.62, , *p <* 1e-6 . **B.** Same but for the repeated rater, ratee = Other block. *r =* .56, *p <* 1e-6.


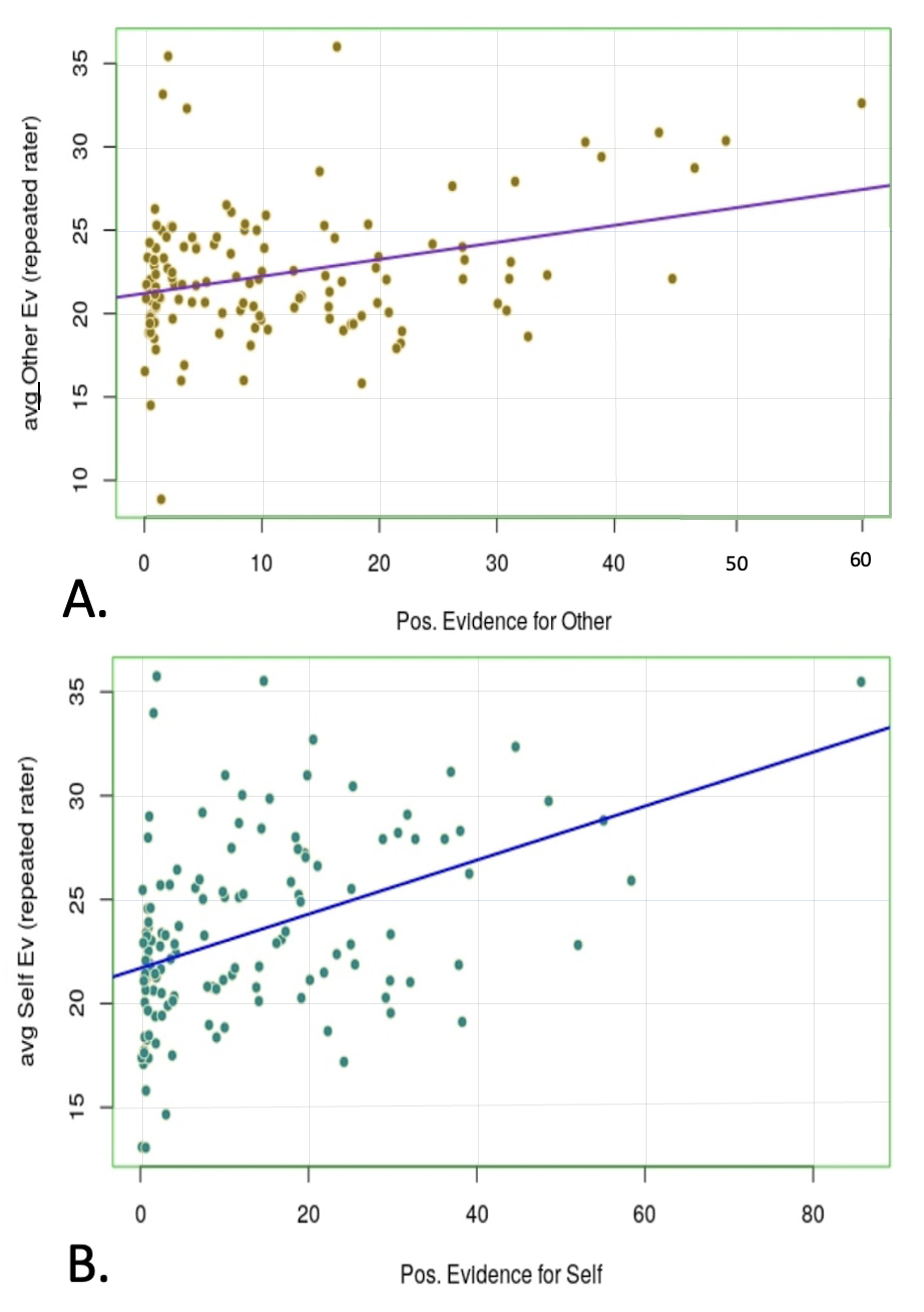


***Fig. S5.*** Replication of published finding, that person-evaluation is modeled by schema positive evidence about that person. The particularly interesting special case of the repeated-rater block is shown, which was not included in published experiments. **A.** Other-evaluation. p-value for the correlation = 0.0014. **B.** Self-evaluation *p =* 2.2 E-7. The schema evidence (*abscissa*) is derived by multiplying the proportion posiSelf (or Other) with 1 + *dEvSelf* (or Other), after transforming the parameters back to native space.


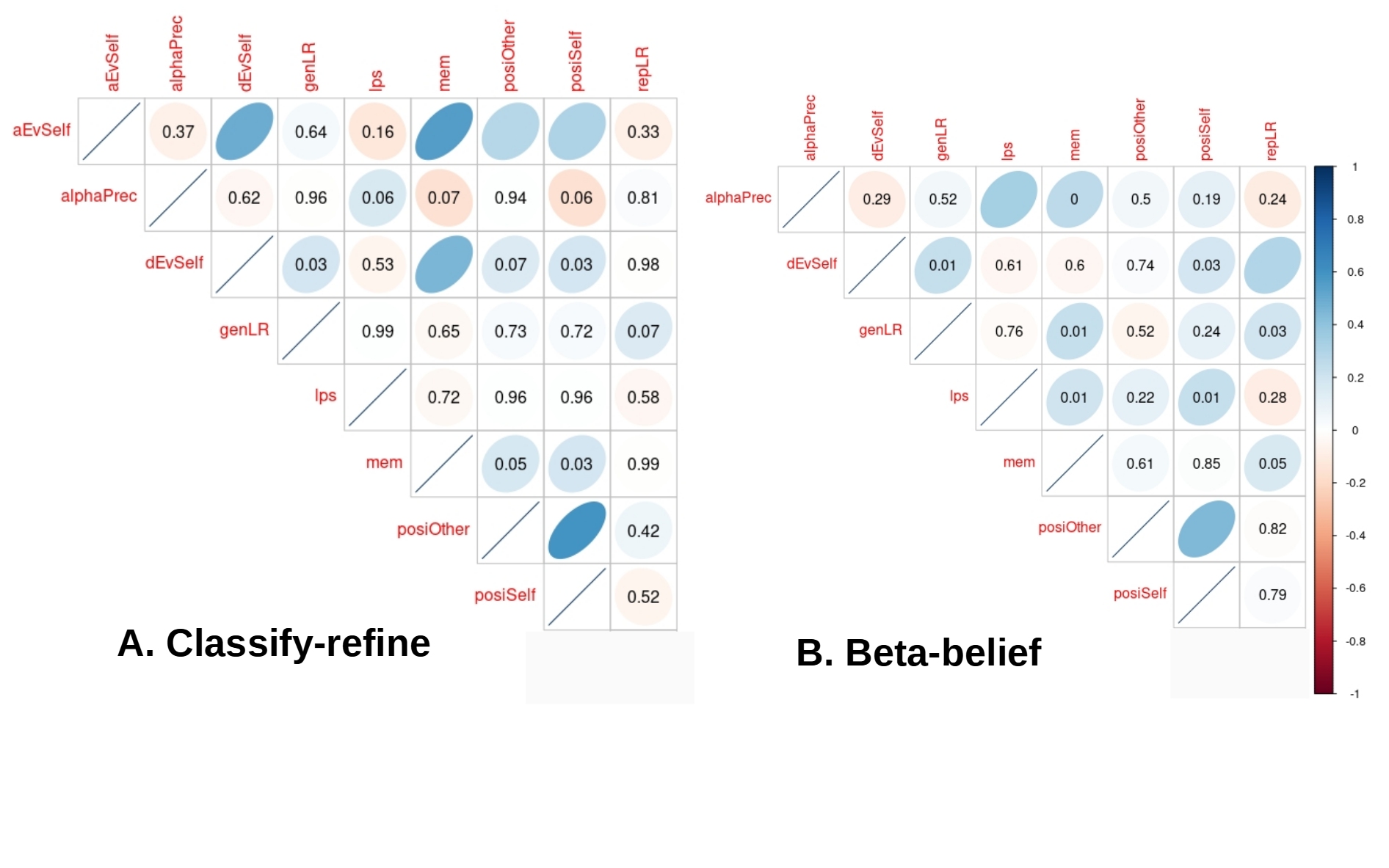


***Fig. S6***. Posterior correlations of the models winning comparisons in the classify-refine and beta-belief classes. Conceptually similar parameters have the same names and corresponding rows in **A.** and **B.** ‘Thermometer’ colors (and ellipsoids girth) denote raw correlation (*blue*) or anticorrelation (*red*) values. The numbers are the Bonferroni-corrected p-values for the correlation coefficients, with no values (i.e., empty cells) meaning *p <* 0.0005 corrected, for clarity. Note that despite most parameters having close conceptual relatives across models, only a few parameter correlations are replicated, notably the positive correlation between *posiSelf* and *posiOther*, and the two confidence parameters, *dEvSelf* and *aEvSelf*, which make psychological sense. This indicates that most correlations do not have a robust psychological meaning. **A.** Classify-refine. Note correlations for *aEvSelf*, the confidence in the likelihood mapping, and memory parameter, mem. **B.** Beta-belief model. Please note that memory parameters have significant correlations, with different parameters in the two models. We tested models fixing or eliminating these, but these were lost in model comparisons.

**2.6 Parameter recovery results**

To carry out parameter recovery, we conducted simulations and refitted data generated to our ‘*classify-refine*’ model used. Parameter recovery revealed strong correlations between fitted and recovered parameters, particularly for key parameters central to our hypotheses. The key parameters are the 1) baseline positivity of raters about self and other (*pS+*, *pO+*), 2) certainty of prior belief of positive attribution (*dEv*), 3) initial certainty parameter for the policy followed by a ‘positive’ or ‘negative’ rater (*aEv*), and 4) decision precision (*αPrec*) were recovered very well, fitted and recovered parameters (Pearson’s *r =* 0.88 to 0.97, see Fig S7A to Fig. S7F).


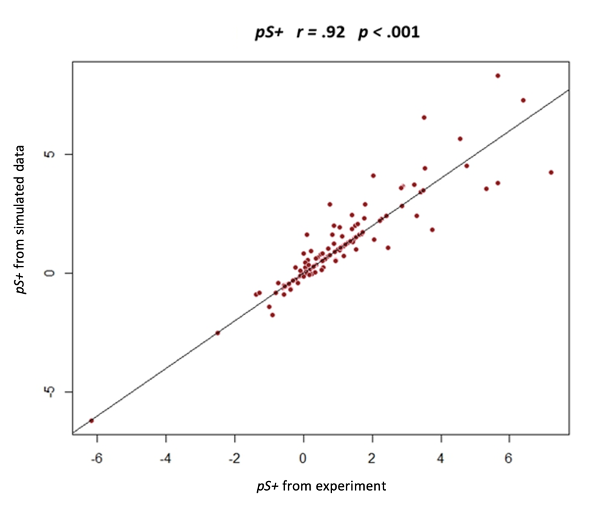


***Fig. S7A.*** Excellent recovery of the ‘positivity of raters about the self’ (*pS+*) parameter.


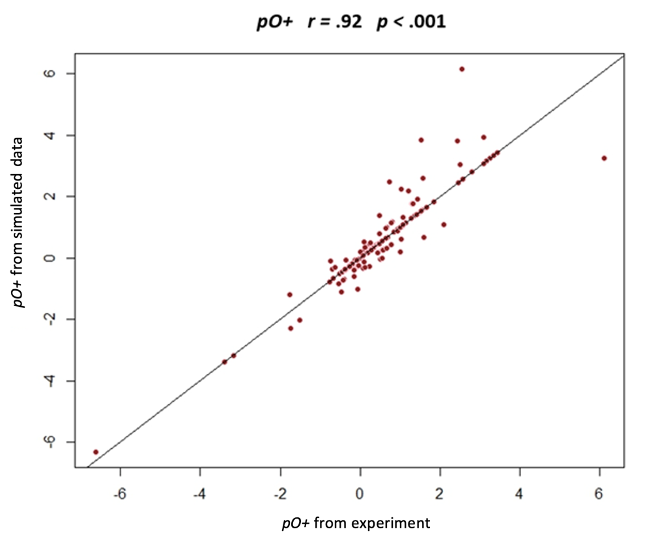


***Fig. S7B.*** Excellent recovery of the ‘positivity of raters about the other’ (*pO+*) parameter.


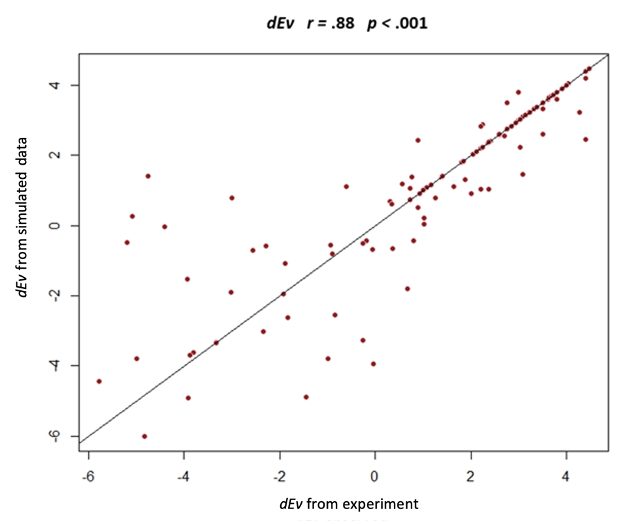


***Fig. S7C***. Good recovery of the ‘certainty of prior belief of positive attribution ’ (*dEv*) parameter.

***
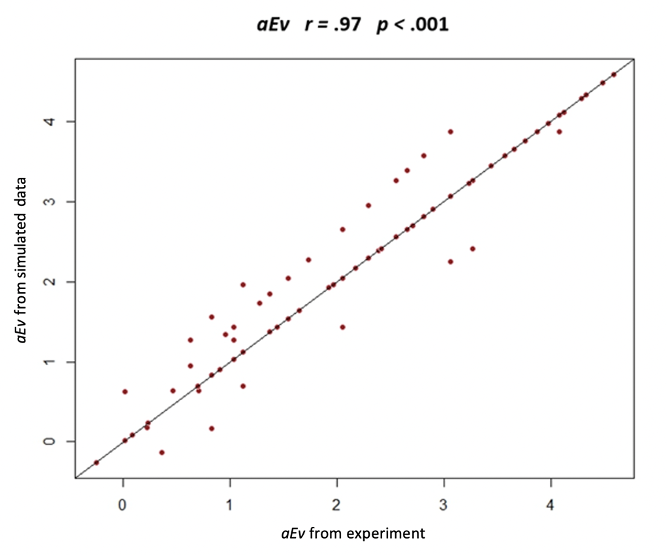
***

***Fig. S7D***. Excellent recovery of the ‘initial certainty parameter for the policy followed by a ‘positive’ or ‘negative’ rater’ (*aEv*) parameter. The discretization of high values is an artifact of the grid used for fitting.


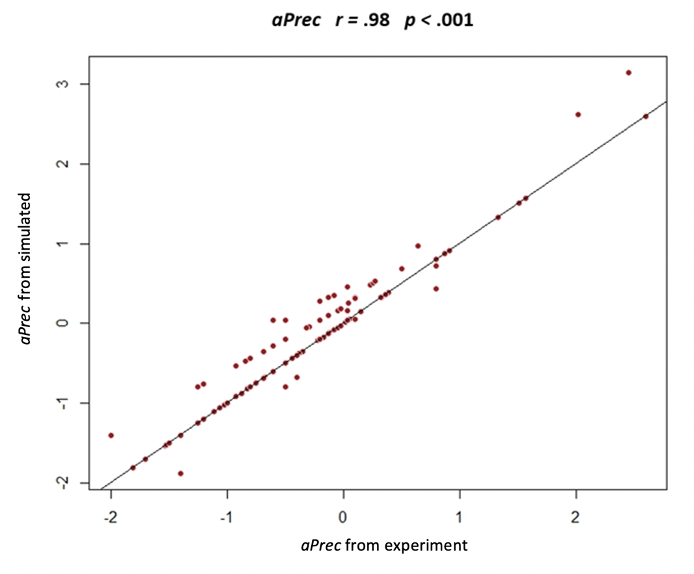


***Fig. S7E.*** Excellent recovery of the ‘decision precision’ (*aPrec*) parameter.

The general learning rate (genLR), memory (*mem*), and between-blocks-of-same-rater learning rate(repLR) parameters Pearson’s *r* for recovery ranged from 0.77 to 0.72. The p-values for N=136 were negligible (*p <*.001).


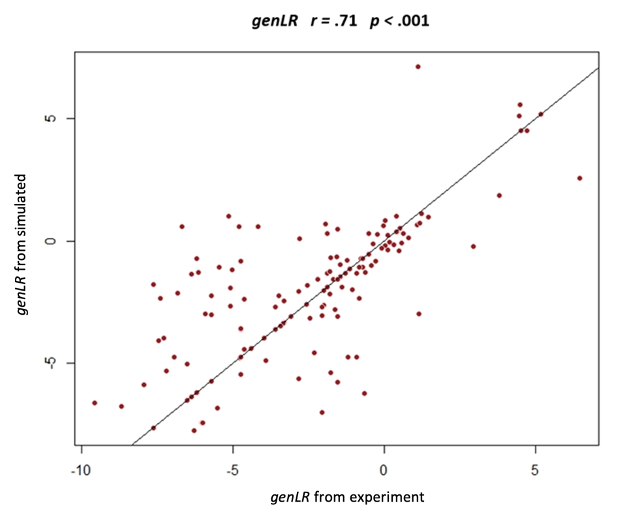


***Fig. S7F.*** Good recovery of the ‘learning rate from rater to rater’ (*genLR*) parameter.


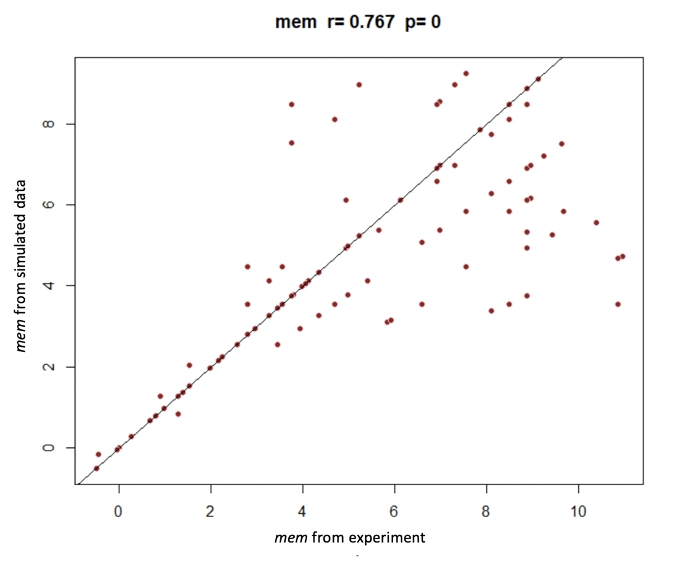


***Fig. S7G.*** Good recovery of the ‘memory’ (*mem*) parameter.


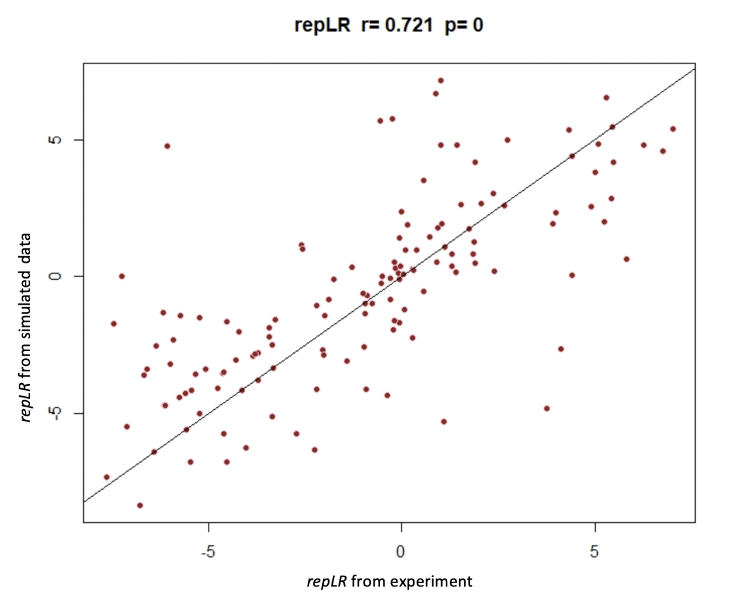


***Fig. S7H*.** Good recovery of the ‘between-blocks-of-same-rater learning rate parameters’ (*repLR*) parameter.

The only parameter that could not be recovered was the level of background noise (*lps*) (N=136), *r =* 0.02, *p =* 0.80. Technically, we note that the transformation of parameters rendered distributions roughly Gaussian, allowing for the use of Pearson's correlations. However, the adaptive grid procedure led to some participants having recovered parameters with the same values as the originally fitted ones, which should not be interpreted as a perfect recovery for these participants.


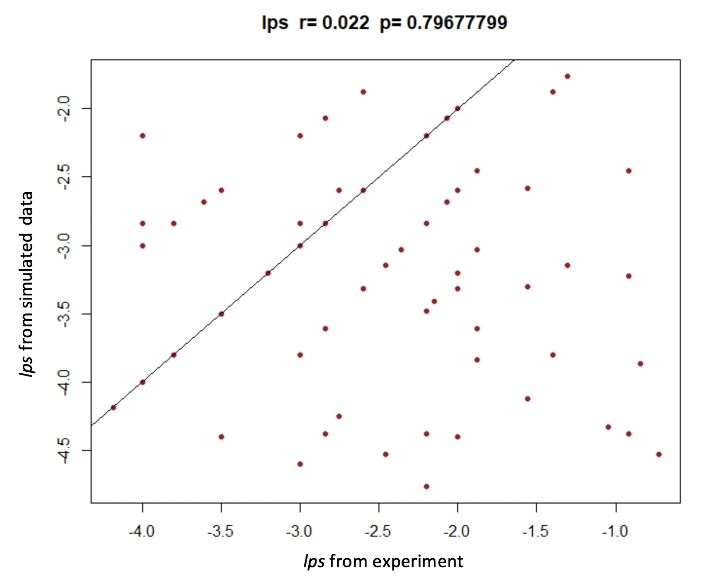


***Fig. S7I.*** Poor recovery of the ‘level of background noise’ (*lps*) parameter.

In conclusion, while we acknowledge the importance of parameter recovery analysis, we believe that in the context of our study, the external validity of parameter estimates is more important than parameter recovery per se. Additionally, we found no evidence that poor recovery of parameters may put the interpretation of the relationships between parameters and mental health measures in question. We are confident that our research provides valuable insights into understanding human behavior and its implications for mental health outcomes.

# Technical notes

Due to randomization issues associated with the combined use of JavaScript *slice()* and *_.shuffle()* functions, some of self-/ other-referential “neutral” blocks were duplicated and missed the non-duplicate “neutral” condition. For behavioral analysis, we treat missing blocks as NA. For modeling, we evaluate the first block of the duplicated blocks of responses for duplicated blocks for each participant. Although the duplication/missing of some “neutral” blocks in a proportion of participants might affect behavioral data analysis, it did not affect the overall modeling of the data as the computational modeling does not necessitate balanced blocks.

# References

[1. Smith R, Friston KJ, Whyte CJ. A step-by-step tutorial on active inference and its application to empirical data. J Math Psychol [Internet]. 2022 Apr 1 [cited 2023 Mar 10];107:102632. Available from: https://www.sciencedirect.com/science/article/pii/S0022249621000973](https://www.zotero.org/google-docs/?02FVOO)

[2. Hopkins AK, Dolan R, Button KS, Moutoussis M. A Reduced Self-Positive Belief Underpins Greater Sensitivity to Negative Evaluation in Socially Anxious Individuals. Comput Psychiatry [Internet]. 2021 Apr 28 [cited 2021 Nov 3];5(1):21–37. Available from: http://www.cpsyjournal.org/article/10.5334/cpsy.57/](https://www.zotero.org/google-docs/?02FVOO)

[3. Hoffmann J, Hobbs C, Moutoussis M, Button K. Lack of optimistic biases in depression and social anxiety is reflected in reduced positive self-beliefs, but distinct processing of social feedback [Internet]. PsyArXiv; 2023 [cited 2023 Oct 12]. Available from: https://osf.io/preprints/psyarxiv/h6ety/](https://www.zotero.org/google-docs/?02FVOO)

[4. Moutoussis M, Hopkins AK, Dolan RJ. Hypotheses About the Relationship of Cognition With Psychopathology Should be Tested by Embedding Them Into Empirical Priors. Front Psychol [Internet]. 2018 [cited 2020 Jul 10];9. Available from: https://www.frontiersin.org/articles/10.3389/fpsyg.2018.02504/full](https://www.zotero.org/google-docs/?02FVOO)

[5. Murphy KP. Machine learning: a probabilistic perspective. Cambridge, MA: MIT press; 2012.](https://www.zotero.org/google-docs/?02FVOO)

[6. Moutoussis M, Bullmore E, Goodyer I, Peter F, Peter J, Dolan R, et al. Change, stability, and instability in the Pavlovian guidance of behaviour from adolescence to young adulthood. PLOS Comput Biol. 2018 Dec 31;14(12):1–26.](https://www.zotero.org/google-docs/?02FVOO)

[7. Prolific [Internet]. London, UK: Prolific; 2023. Available from: https://www.prolific.com/](https://www.zotero.org/google-docs/?02FVOO)
